# RaGOO: fast and accurate reference-guided scaffolding of draft genomes

**DOI:** 10.1186/s13059-019-1829-6

**Published:** 2019-10-28

**Authors:** Michael Alonge, Sebastian Soyk, Srividya Ramakrishnan, Xingang Wang, Sara Goodwin, Fritz J. Sedlazeck, Zachary B. Lippman, Michael C. Schatz

**Affiliations:** 1 Department of Computer Science, Johns Hopkins University, Baltimore, MD USA; 2 Cold Spring Harbor Laboratory, Cold Spring Harbor, NY USA; 3 Human Genome Sequencing Center, Baylor College of Medicine, Houston, TX USA; 4 Cold Spring Harbor Laboratory, Howard Hughes Medical Institute, Cold Spring Harbor, NY USA; 5 Department of Biology, Johns Hopkins University, Baltimore, MD USA

**Keywords:** Pseudomolecule, Reference-guided, Genome assembly, Scaffolding, Genome alignment, Long-read sequencing, Tomato

## Abstract

We present RaGOO, a reference-guided contig ordering and orienting tool that leverages the speed and sensitivity of Minimap2 to accurately achieve chromosome-scale assemblies in minutes. After the pseudomolecules are constructed, RaGOO identifies structural variants, including those spanning sequencing gaps. We show that RaGOO accurately orders and orients 3 de novo tomato genome assemblies, including the widely used M82 reference cultivar. We then demonstrate the scalability and utility of RaGOO with a pan-genome analysis of 103 *Arabidopsis thaliana* accessions by examining the structural variants detected in the newly assembled pseudomolecules. RaGOO is available open source at https://github.com/malonge/RaGOO.

## Background

Long-read single-molecule sequencing technologies commercialized by Oxford Nanopore Technologies (ONT) and Pacific Biosciences (PacBio) have facilitated a resurgence of high-quality de novo eukaryotic genome assemblies [1]. Assemblies using these technologies in a variety of plant and animal species have consistently reported contig N50s over 1 Mbp, while also reconstructing higher percentages of target genomes, including repetitive sequences [2, 3]. Current long-read sequencers are now able to produce over one terabase of long reads per week, presenting the opportunity for detailed pan-genome analysis of unprecedented scale. Such analyses can include structural variations that are notoriously difficult to detect using short-read sequencing. However, lagging behind the current speed and cost of generating long-read sequencing data are genome assemblers, which are still unable to resolve complex repeats and related structural variants that are widespread in eukaryotic genomes. Thus, there is a need for simplified and faster approaches to scaffold fragmented genome assemblies into chromosome-scale pseudomolecules.

Two common approaches have been used to achieve chromosome-scale assemblies, namely, reference-free (de novo) and reference-guided approaches. One popular reference-free scaffolding approach is to anchor genome assembly contigs to some variety of genomic map [4], such as an optical, physical, or linkage map [5]. This process involves aligning the genomic map to a sequence assembly and scaffolding contigs according to the chromosomal structure indicated in the map. However, contigs not implicated in any alignments will fail to be scaffolded, which can result in incomplete scaffolding. Furthermore, acquiring a genomic map can be expensive, time-consuming, or otherwise intractable depending on the species and the type of map.

Another reference-free method for pseudomolecule construction involves the use of long-range genomic information to scaffold assembled contigs. This includes a large class of technologies such as mate-pair sequencing, Bacterial Artificial Chromosomes (BACs), Linked Reads and chromatin conformation capture such as Hi-C [6–8]. In particular, Hi-C has recently been shown to be a practical and effective resource for chromosome-scale scaffolding [9–11]. Paired-end Hi-C sequencing reads are aligned to the assembly, and mates which align to different contigs (Hi-C links) are recorded. According to the relative density of such Hi-C links between pairs of contigs, contigs can be ordered and oriented into larger scaffolds, potentially forming chromosome-length pseudomolecules. Also, because misassemblies may be observed by visualizing Hi-C alignments, Hi-C can be used for validation and manual correction of misassemblies [12]. Though Hi-C has been widely adopted, there remain challenges that can impede the ability to form accurate chromosome-scale pseudomolecules with Hi-C alone. Principally, Hi-C data are noisy, and Hi-C-based scaffolders are prone to producing structurally inaccurate scaffolds [13]. Also, because this process relies on the alignment of short Hi-C sequencing reads to the draft assembly, small and repetitive contigs with little or conflicting Hi-C link information often fail to be accurately scaffolded. Finally, the analysis requires deep sequencing coverage and therefore can be expensive and compute-intensive.

Aside from reference-free approaches, there are also a few tools available for reference-guided scaffolding [14]. For example, Chromosomer and MUMmer’s “show-tiling” utility leverage pairwise alignments to a reference genome for contig scaffolding and have been used to scaffold eukaryotic genomes [15–18]. RACA is similar, though it also requires paired-end sequencing data to aid scaffolding [19]. Finally, tools such as GOS-ASM and Ragout2 employ multiple sequence aligners to reconcile multiple, potentially diverse contig sets [20, 21]. Though reference-guided scaffolding may introduce erroneous reference bias, it is often substantially faster and less expensive than acquiring the resources for the reference-free methods outlined above. However, current tools for reference-guided scaffolding of eukaryotic genomes have notable shortcomings. Firstly, these tools depend on slower DNA aligners such as BLAST and Nucmer and accordingly require long compute times of several hours to several days for mammalian-sized genomes [22]. This is especially pronounced in tools like Ragout2 that use multiple sequence aligners, such as Cactus, that can require hundreds of CPU hours for large eukaryotic genomes [23]. These aligners are also not robust to repetitive and/or gapped alignments resulting in a significant portion of contigs being unassigned in pseudomolecules. Finally, many of these methods do not internally offer the ability to correct large-scale misassemblies frequently present in draft assemblies of eukaryotic genomes nor report any metrics on conflicts due to true biological differences in the genomes.

Here, we introduce RaGOO, an open-source method which utilizes Minimap2 [24] alignments to a closely related reference genome to quickly cluster, order, and orient genome assembly contigs into pseudomolecules. RaGOO also provides the option to correct apparent chimeric contigs prior to pseudomolecule construction. Finally, structural variants (SVs), including those spanning gap sequence, are identified using an optimized and integrated version of Assemblytics [25], thus enabling rapid pan-genome SV analysis of many genomes at once. This is especially important for detecting large insertions and other complex structural variations that are difficult to detect using read mapping approaches.

We first demonstrate the speed and accuracy of RaGOO scaffolding with simulated data of increasing complexity and show that it outperforms 2 popular alternative methods. We next show the utility of RaGOO by creating high-quality chromosome-scale reference genomes for 3 distinct wild and domesticated genotypes of the model crop tomato using a combination of short and long-read sequencing. Finally, we demonstrate the scalability of RaGOO by ordering and orienting 103 draft *Arabidopsis thaliana* genomes and comparing structural variants across the pan-genome. This uncovers a large number of defense response genes that are highly variable.

## Results

### Reference-guided contig ordering and orientation with RaGOO

**Fig. 1:**
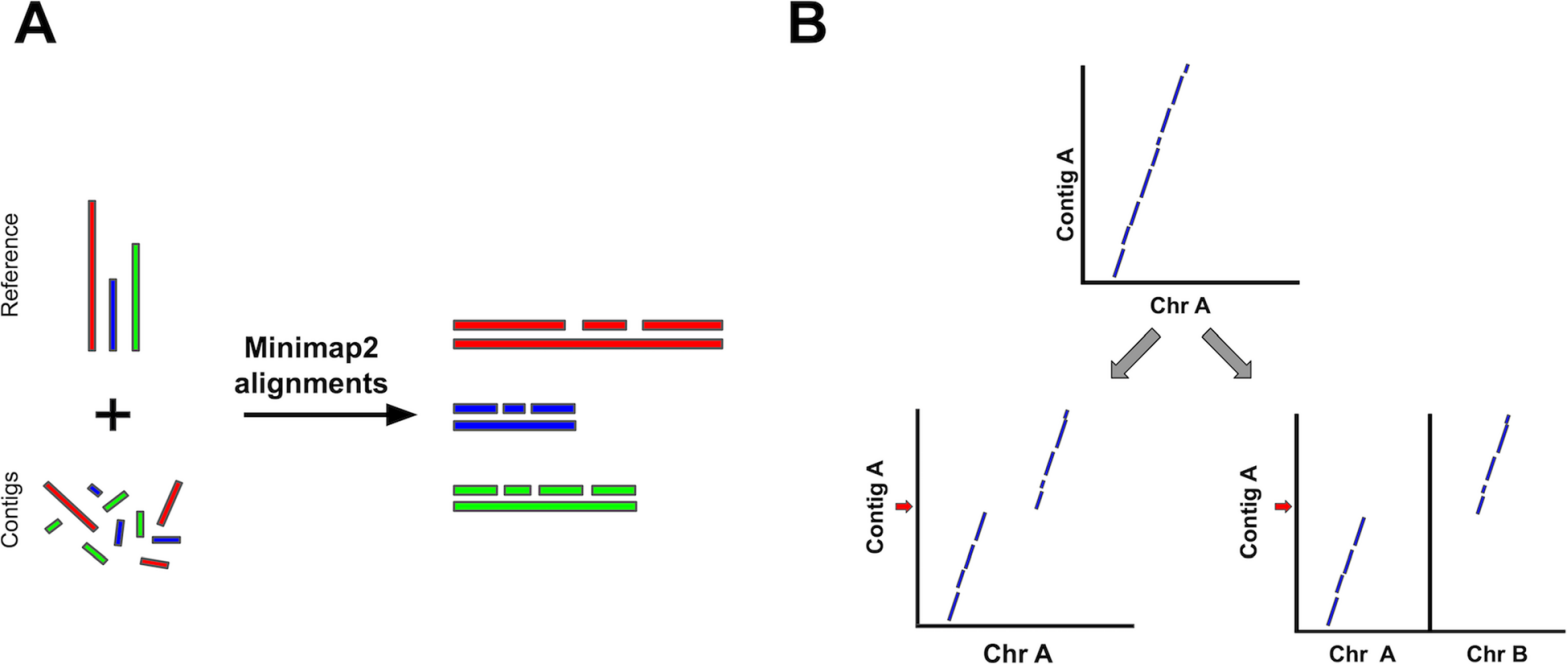
The RaGOO pipeline. **a** Contigs are aligned to the reference genome with Minimap2 and are ordered and oriented according to those alignments. **b** Normal alignments between a contig and a reference chromosome (top) and example alignments between a reference chromosome and an intrachromosomal chimera (bottom left) and an interchromosomal chimera (bottom right). Red arrows represent potential contig breakpoints

RaGOO is a fast and reliable reference-guided scaffolding method, implemented as an open-source python command-line utility, that orders and orients genome assembly contigs according to Minimap2 alignments to a single reference genome (Fig. 1) [26]. RaGOO’s primary goal is to utilize the large-scale structure of a reference genome to organize assembly contigs, analogous to how a genetic map is used. Therefore, under default settings, RaGOO does not alter or mutate any input assembly sequence but rather arranges them and places gaps for padding between contigs. Additionally, users have the option to break input contigs at points of potential misassembly indicated by discordant alignments to the reference genome. However, these breaks will only fragment the assembly and do not add or remove any sequence content. RaGOO can optionally avoid breaking chimeric intervals at loci within genomic coordinates specified by a gff3 file, so as to avoid disrupting gene models identified in the de novo assembly.

Additionally, RaGOO computes confidence scores associated with the clustering, ordering, and orienting of each contig (the “Methods” section). These scores ultimately strive to measure the fidelity of contig ordering and orienting to the underlying alignments. For example, a contig which aligns with equal coverage to three different chromosomes will have a lower clustering confidence score than a contig which exclusively aligns to a single chromosome. These scores can also be viewed as measuring the level of scaffolding ambiguity present in the alignments. Accordingly, one can compare confidence scores with and without chimeric contig correction to ensure that alignments become less ambiguous after correction (see the “M82 chromosome Hi-C validation and finishing and annotation” section). Furthermore, a poor confidence score distribution can indicate that a draft assembly is too divergent from the reference assembly for optimal scaffolding (see the “Scaffolding a divergent *S. pennellii* genome assembly” section).

After constructing pseudomolecules, RaGOO re-aligns the assembly to the reference and calls structural variants with an integrated version of Assemblytics. We have optimized this approach by replacing the relatively slow single-threaded nucmer alignment phase with the much faster Minimap2 aligner along with the necessary converters between the output formats. Noting that such alignments may traverse gaps in either the reference or the query assembly, we report the percent overlap between each SV and gaps, allowing users to utilize such variants at their discretion. Importantly, the speed of Minimap2 alignments, and therefore RaGOO, facilitates a genome scaffolding and SV analysis at scales previously not feasible with comparable tools. For example, RaGOO scaffolds an *Arabidopsis thaliana* draft assembly in ~ 13 s and a human draft assembly in ~ 12 min and 33 s using eight cores and less than 20 GB of RAM (Additional file 1: Figure S1) [27].

### Simulated reference-guided scaffolding

To assess the efficacy of RaGOO, we used it to scaffold simulated draft eukaryotic genome assemblies of increasing difficulty. To simulate these assemblies, we partitioned the current tomato (*Solanum lycopersicum*) reference genome (Heinz version SL3.0) into variable-length scaffolds [28]. To achieve a realistic distribution of sequence lengths, we sampled the observed contig lengths from a de novo assembly produced with Oxford Nanopore long reads of the *S. lycopersicum* cultivar M82, which is described later in this paper (the “Methods” section). Given that many of these resulting scaffolds contained a gap sequence ("N" characters) from the reference genome, we also established an assembly comprised of contigs free of sequencing gaps. For this, we split the simulated scaffolds at any stretch of 20 or more “N” characters, excluding the gap sequence. We also excluded any resulting contigs shorter than 10 kbp in length. We refer to these scaffolds and contigs as the “easy” set of simulated data, as they are a partitioning of the reference with no variation. To simulate a “hard” dataset that contained variation, we used SURVIVOR [29] to simulate 10,000 insertion and deletion SVs, ranging in size from 20 bp to 10 kbp in size, and SNPs at a rate of 1% into the simulated scaffolds. Contigs were then derived from these scaffolds just as with the “easy” contigs. Assembly stats for these 4 simulated assemblies are in Additional file 1: Table S1.

Utilizing the same SL3.0 reference assembly, we used MUMmer’s “show-tiling” utility, as well as Chromosomer and RaGOO to arrange these simulated assemblies into 12 pseudomolecules. To assess scaffolding success, we measured clustering, ordering, and orienting accuracy. Clustering and orienting accuracy is the percentage of localized contigs that were assigned the correct chromosome group and orientation, respectively. To assess the ordering accuracy, the edit distance between the true and predicted contig order was calculated for each pseudomolecule normalized by the true number of contigs in the pseudomolecule. Additionally, for a local measurement of ordering accuracy, the fraction of correct adjacent contig pairs was computed for each pseudomolecule. Finally, to measure the scaffolding completeness, we noted the percentage of contigs and total sequence localized into pseudomolecules.

**Fig. 2:**
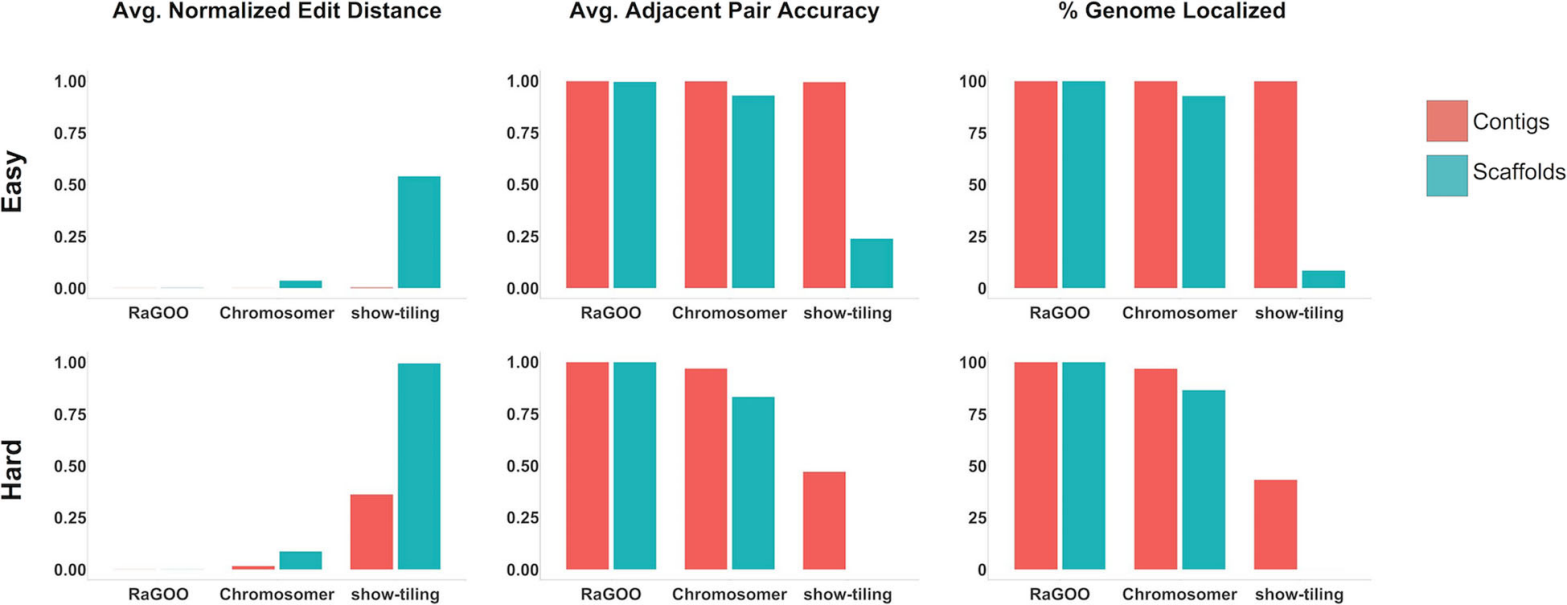
Scaffolding simulated assemblies. Ordering and localization results for “easy” and “hard” simulated tomato genome assemblies. Normalized edit distance and adjacent pair accuracy measure the success of contig ordering and are averaged across the 12 simulated chromosomes. The percentage of the genome localized measures how much of the simulated assemblies were clustered, ordered and oriented into pseudomolecules

RaGOO performed best on all datasets, achieving high clustering, ordering, and orienting accuracy on both the “easy” and “hard” datasets, while localizing the vast majority (~ 99.9998% for hard scaffolds) of sequence in only a few minutes (1 min and 15 s for the “hard” scaffolds) (Fig. 2, Additional file 2: Table S2). In all simulations, Chromosomer accurately reconstructed most of the genome, though the presence of gaps in scaffolds and variation in the “hard” assembly degraded the performance to a localization score of 86.65% in the “hard” scaffolds. Show-tiling suffered tremendously from the presence of gaps in scaffolds and accordingly achieved poor localization scores on scaffolds of both the “easy” (8.43%) and “hard” (0.01%) sets. Both Chromosomer and show-tiling took substantially longer to run than RaGOO in all cases and required several hours rather than minutes.

### Pan-SV analysis of three chromosome-scale tomato genome assemblies

For more than a decade, the reference genome for tomato (var. “Heinz 1706”) has been an invaluable resource in both basic and applied research, but extensive sequence gaps (81.7 Mbp, 9.87%), unlocalized sequence (~ 17.8 Mbp, 2.39%), and limited information on natural genetic variation in the wider germplasm pool impeded its full utilization [28]. To compensate, more than 700 additional accessions have since been sequenced by Illumina short-read technology [30, 31]. However, due to the short sequence reads, these studies were limited to evaluating, with reasonable accuracy (depending on variable sequencing quality and coverage), single nucleotide polymorphisms (SNPs) and small insertions and deletions (indels). In contrast, larger structural variations (SVs) that have important and often underestimated functional consequences for genome evolution and phenotypic diversity were largely ignored in this major model crop plant. Critically, without long reads, the complete catalog of structural variations in the species, a pan-SV analysis, is largely incomplete.

To address this knowledge gap and begin constructing a high-quality tomato pan-SV analysis, we used long-read ONT instruments to sequence three distinct genotypes that provide anchor points for wild and domesticated tomato germplasm: (1) the species *S. pimpinellifolium* is the ancestor of tomato, and the Ecuadorian *S. pimpinellifolium* accession BGV006775 (BGV) represents the group of progenitors that are most closely related to early domesticated types; (2) the *S. lycopersicum* processing cultivar M82 is the most widely used accession in research due to its rich genetic resources; and (3) the *S. lycopersicum* elite breeding line Fla.8924 (FLA) is a large-fruited “fresh market” type that was developed for open-field production in Florida [32, 33]. Together, these three accessions provide a foundation for constructing a pan-SV analysis that will enable the identification and classification of thousands of predicted SVs.

#### Reference-guided and reference-free M82 scaffolding

**Fig. 3:**
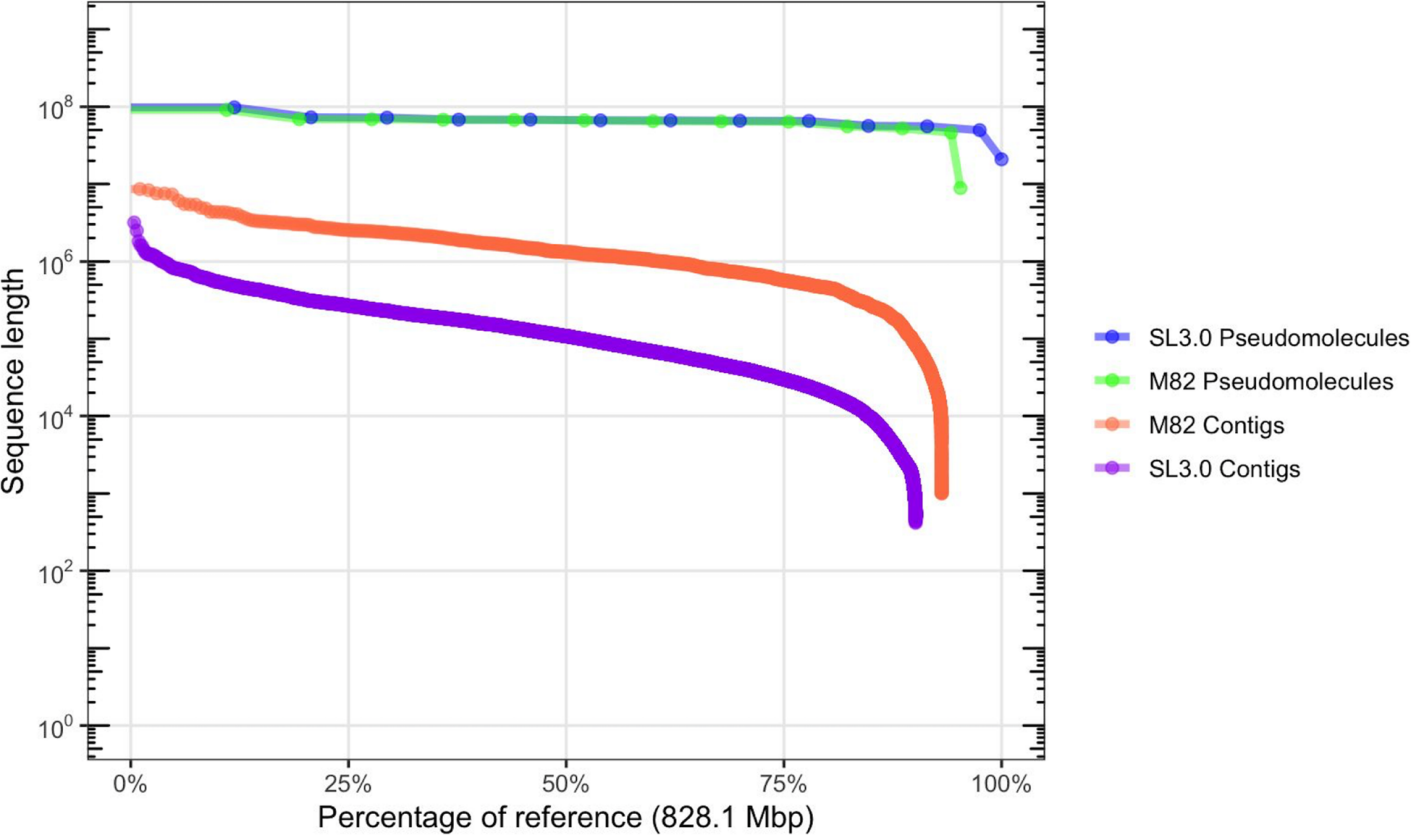
M82 assembly contiguity. “Nchart” of the M82 and Heinz contigs and pseudomolecules. M82 pseudomolecules were established by ordering and orienting M82 contigs with RaGOO. Heinz contigs were derived from the SL3.0 pseudomolecules by splitting sequences at stretches of 20 or more contiguous “N” characters

In order to evaluate the effectiveness of RaGOO with genuine sequencing data, we first used it along with other reference-guided and reference-free tools to scaffold a highly contiguous assembly of the *S. lycopersicum* cultivar M82. We sequenced the genome with an Oxford Nanopore MinION sequencer to 58.8× fold coverage with an N50 read length of 13.4 kbp (max 1,256,650 bp). The genome was assembled with Canu [34] and was comprised of 1709 contigs with a contig N50 of 1,458,445 bp. To compare RaGOO to other reference-guided tools, the assembly was scaffolded with RaGOO (with chimeric contig correction), MUMmer’s “show-tiling” utility, and Chromosomer. Here, a “localized” contig is one that is placed in a pseudomolecule group and is assigned order and orientation. In all cases, the Heinz SL3.0 genome was used as the reference. RaGOO localized the highest portion of sequence, placing 99.01% of sequence into chromosomes compared to 85.6% and 3.17% for Chromosomer and show-tiling, respectively (Additional file 1: Table S3). The resulting RaGOO assembly contained 12 chromosome-length pseudomolecules with only 0.99% of sequence in the ambiguous chromosome 0 (Fig. 3, Additional file 1: Figure S2). Additionally, the scaffolding completed in only ~ 3 min for RaGOO, compared to ~ 285 min for show-tiling and ~ 1466 min for Chromosomer.

To compare RaGOO scaffolding to a widely used reference-free approach, we generated Hi-C chromatin conformation data and used SALSA2 [13] to build scaffolds from the M82 contigs. Though SALSA2 does not necessarily build pseudomolecules, it strives to establish chromosome and chromosome-arm length scaffolds as the data allows. SALSA2 utilized Hi-C alignments to the M82 draft assembly along with the M82 Canu assembly graph. Though the scaffolds were highly contiguous compared to the input assembly (scaffold N50 of 18,282,950 bp), they fall far short of complete chromosome scale.

**Fig. 4:**
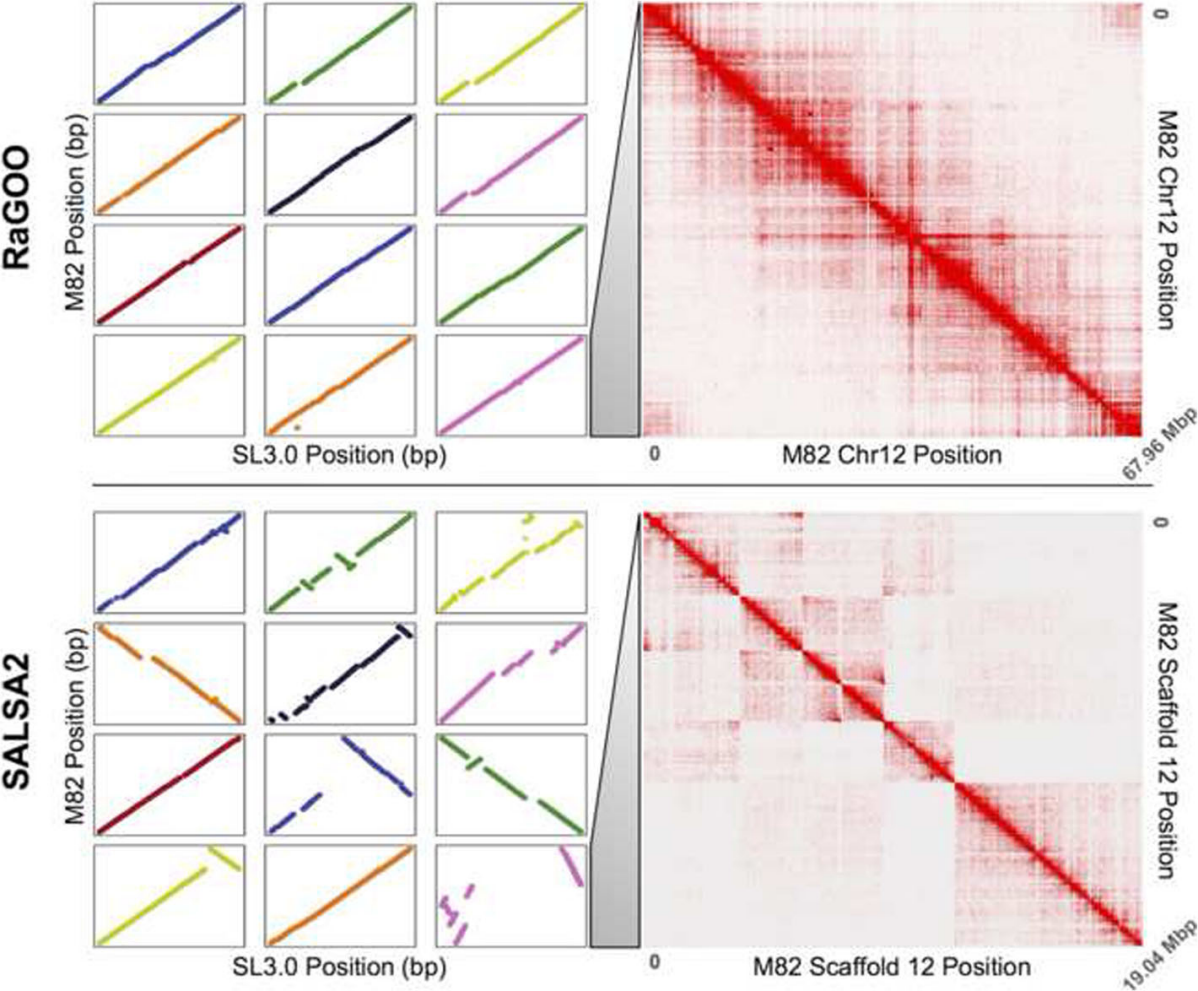
Reference-free vs. reference-guided scaffolding of M82. Both the top and bottom panels depict a dotplot (left) and Hi-C heatmap (right). The dotplots are generated from alignments to the Heinz reference assembly. On the top panel is the reference-guided RaGOO assembly dotplot, with chromosomes 1 through 12 depicted from top left to bottom right, and the Hi-C heatmap for chromosome 12. On the bottom is the de novo SALSA scaffolds dotplot, with the 12 largest scaffolds depicted in descending order of length from top left to bottom right and the Hi-C heatmap for the 12th largest scaffold

We further compared the structural accuracy of the RaGOO pseudomolecules to that of the SALSA2 scaffolds by comparing the 12 pseudomolecules of the former and the 12 longest scaffolds of the latter to the Heinz SL3.0 reference. The dotplots from these alignments are displayed in Fig. 4 (left). This shows nearly complete and highly co-linear coverage of the RaGOO pseudomolecules, while highly fragmented and rearranged placements of the SALSA2 scaffolds. Additionally, realigning the same Hi-C data to these pseudomolecules/scaffolds provides a reference-free assessment of the large-scale structural accuracy of these sequences. Through this analysis, we found that the SALSA2 scaffolds contained many misassemblies, especially false inversions, while the RaGOO pseudomolecules contained very few structural errors (Fig. 4 right). These Hi-C alignments suggest that most inversions and other large structural differences between the SALSA2 scaffolds and the Heinz reference assembly are likely not biological, but rather are scaffolding errors. They also demonstrate that erroneous reference bias in the RaGOO pseudomolecules, though present, was rare.

#### M82 chromosome Hi-C validation and finishing and annotation

In an effort to establish a new structurally accurate tomato reference genome, we sought to make further improvements to the RaGOO M82 pseudomolecules, as they provided the best completeness and contiguity with relatively few misassemblies. We first used the abovementioned Hi-C data and Juicebox Assembly Tools to correct apparent lingering misassemblies in the pseudomolecules [12]. A total of three corrections were made: an inversion error correction on chromosome 3 and an ordering error correction on chromosomes 7 and 11. Any “debris” contigs resulting from these alterations were placed in chromosome 0. With these few misassemblies corrected, the pseudomolecules were gap filled with PBJelly and polished with Pilon [35, 36] (the “Methods” section, Additional file 3: Table S4). The final polished assembly had an average identity of 99.56% when comparing to the Heinz SL3.0 reference and contained a complete single copy of 94.1% of BUSCO genes [37]. We note that M82 is biologically distinct from Heinz, so we do not expect 100% identity and estimate the overall identity at approximately 99.8 to 99.9%. Additionally, M82 consensus accuracy is reflected in ITAG 3.2 cDNA GMAP alignments, 96.8% of which align with at least 95% coverage and identity (Additional file 1: Figure S3) [38].

Gene finding and annotation was performed on the finished M82 assembly with the MAKER pipeline [39] (the “Methods” section, Additional file 1: Figure S4, Additional file 4: Table S5). There are 35,957 genes annotated in the M82 assembly, of which 27,624 are protein coding. When comparing M82 and Heinz 1706 ITAG3.2 gene models using gffcompare (https://github.com/gpertea/gffcompare), we found 24,652 gene models with completely matching intron chains. The final M82 assembly contained a total of ~ 46 Mbp novel non-gapped sequence missing from the SL3.0 reference genome. Furthermore, the M82 assembly contained only ~ 8.9 Mbp of unlocalized sequence in chromosome 0 compared to ~ 17.8 Mbp in the Heinz SL3.0 reference.

#### Pan-SV analysis of 3 tomato accessions

In addition to the M82 cultivar, we also assembled genomes for the BGV and FLA tomato accessions de novo with Oxford Nanopore sequencing reads and the Canu assembler. We sequenced the BGV accession to 33.5× fold coverage with a read N50 length of 27,350 bp (max 192,728 bp) and the FLA accession to 41.6× fold coverage with a read N50 length of 24,225 bp (max 144,350 bp). The FLA assembly contained a total of 750,743,510 bp and had an N50 of 795,751 bp, while the BGV assembly contained a total of 769,694,915 bp and had an N50 of 4,105,177 bp. As with the M82 assembly, RaGOO was then used to establish pseudomolecules and call structural variants for these assemblies. The final FLA and BGV pseudomolecules contained 745,663,382 bp and 765,377,903 bp (99.3% and 99.4%) of the total ungapped sequence localized to chromosomes, respectively. Finally, the assemblies underwent gap filling, polishing, and gene finding using the same methods as M82 (Additional file 1: Table S5). A summary of the final assembly statistics for all three accessions is presented in Table 1. The polished assemblies had 99.4% (FLA) and 98.9% (BGV) average identity compared to the Heinz SL3.0 reference as measured by MUMmer’s “dnadiff.” These assemblies also demonstrated genome completeness with BGV and FLA containing a single copy of 94.8% and 94.9% of BUSCO genes, respectively.

**Table 1:** Summary statistics of the reference tomato genome as well as the three novel accessions. Chromosome span indicates the total span of all of the chromosomes, including gaps. Chromosome N50 is the length such that half of the total span is covered in chromosome sequences this length or longer. Chr0 bases report the number of bases assigned to the unresolved chromosome 0. Contig span is the total length of non-gap (N) characters. Contig N50 is the length such that half of the contig span is covered by contigs this length or longer. Number SVs reports the number of SVs reported by RaGOO using the integrated version of Assemblytics

Accession	Chromosome span (bp)	Chromosome N50 (bp)	Chr0 bases (bp)	Number Contigs	Contig span (bp)	Contig N50 (bp)	Number SVs
Heinz	828,076,956	66,723,567	20,852,292	22,705	746,357,581	133,084	NA
M82	792,934,937	67,021,692	8,891,603	2910	771,143,786	1,458,445	36,191
BGV	794,568,563	67,174,401	4,643,553	638	769,694,915	4,105,177	45,927
FLA	796,004,315	67,650,907	5,490,904	2577	750,743,510	795,751	45,478

**Fig. 5:**
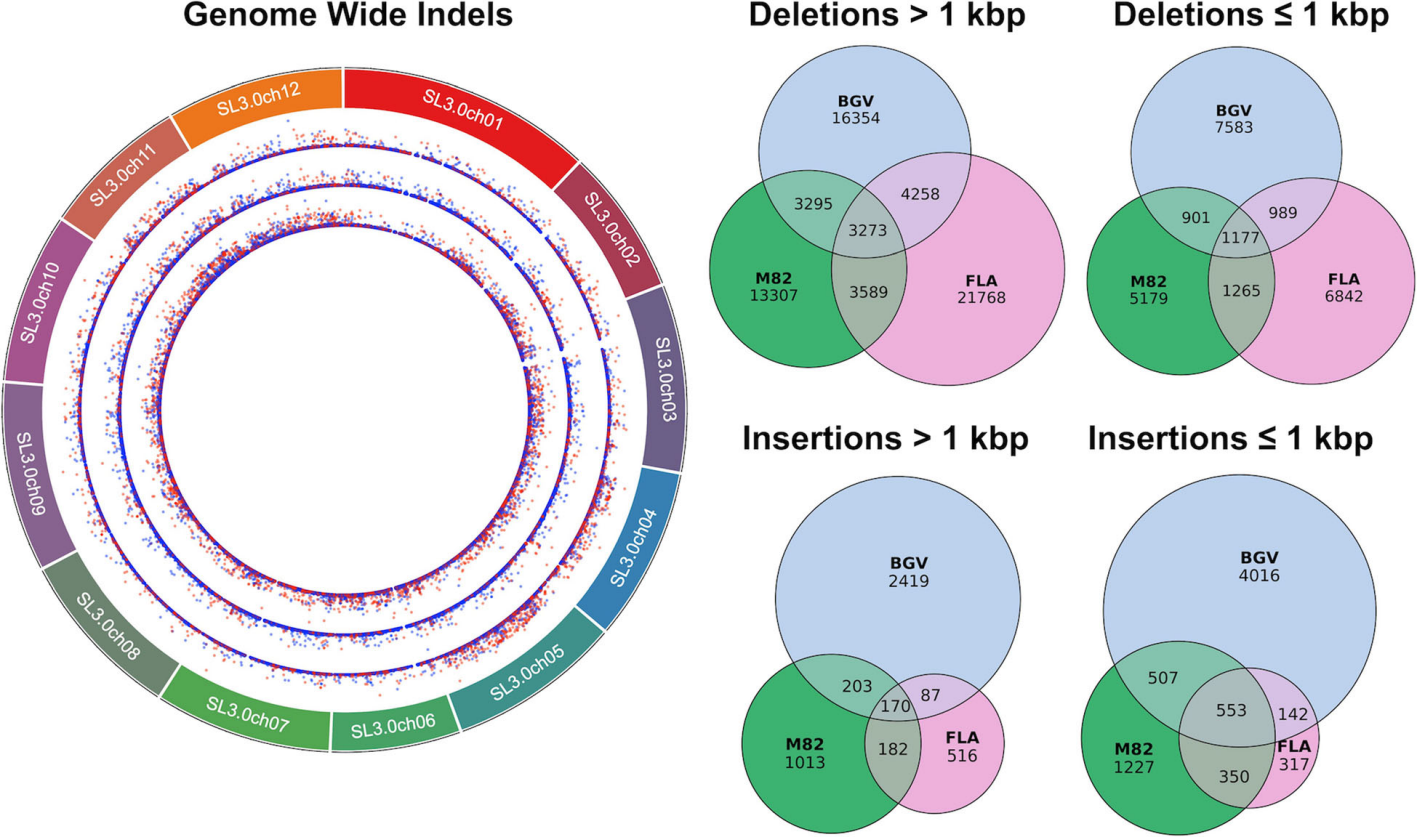
The tomato pan-genome. (left) Circos plot (http://omgenomics.com/circa/) depicting the size and type of structural variant. From the outer ring to the inner ring: M82, FLA, and BGV. Point height (*y*-axis) is scaled by the size of the variant, with red indicating insertions and blue indicating deletions. (right) Euler diagrams (https://github.com/jolars/eulerr) depicting the insertions and deletions shared among the three accessions

Together with the M82 genome, we present 3 chromosome-scale assemblies with substantially more sequence content and fewer gaps than the Heinz SL3.0 reference genome. Given the structural variants output by RaGOO, we next used SURVIVOR to determine which variants were shared among these three accessions (Fig. 5). As expected, the most divergent accession, BGV, demonstrated the most structural variant diversity with a total of 45,927 SVs compared to 45,478 and 36,191 SVs in FLA and M82, respectively. The union of these sets of variants yielded 98,988 total structural variants, which overlapped with 19,790 out of 35,768 total ITAG 3.2 genes (with 2 kbp flanking upstream and downstream each gene included). A complete list of gene/variant intersections is available in Additional file 5: Table S6. The most variable gene (the gene with the most intersecting SVs), Solyc03g095810.3, is annotated as a member of the GDSL/SGNH-like Acyl-Esterase family, while the second most variable gene, Solyc03g036460.2, is annotated as a member of the E3 ubiquitin-protein ligase. These three chromosome-scale assemblies, along with their associated sets of SVs, establish valuable genomic resources for the *Solanaceae* scientific community.

### Scaffolding a divergent *S. pennellii* genome assembly

Reference-guided scaffolding accuracy depends on a shared chromosomal structure between the draft and reference assemblies. This is the case for our three tomato assemblies since they represent either the same species as the reference (*S. lycopersicum*) or a closely related progenitor species (*S. pimpinellifolium*). However, we sought to evaluate the scaffolding success of a more divergent *S. pennellii* draft assembly in order to assess scenarios where assemblies are not close relatives. To this end, we scaffolded a draft *S. pennellii* genome assembly twice using two distinct reference genomes [40]. First, we scaffolded contigs according to the same *S. lycopersicum* SL3.0 reference genome used thus far in our previous tomato analysis. In addition, we also scaffolded contigs according to an independent, chromosome-scale *S. pennellii* reference genome [41].

If the distantly related *S. lycopersicum* reference is suitable for scaffolding the *S. pennellii* contigs, then the two resulting sets of RaGOO pseudomolecules should be structurally similar. Rather, we found major structural disagreements between the two sets of RaGOO pseudomolecules (Additional file 1: Figure S5). Notably, chromosome 0 contained over four times as many bases when using the *S. lycopersicum* reference (26,868,206 bp vs. 6,230,859 bp) indicating that significantly less of the genome had been localized. We further noted the confidence score distributions were appreciably lower when using the *S. lycopersicum* reference (Additional file 1: Figure S6). From these results, we conclude that *S. lycopersicum* is too divergent from *S. pennellii* to be used as a guide for scaffolding. Though every case must be examined individually, this analysis shows how confidence scores and localization stats can be used to determine if reference-guided scaffolding is appropriate for divergent assemblies.

### Pan-SV analysis of 103 *Arabidopsis thaliana* genomes

**Fig. 6:**
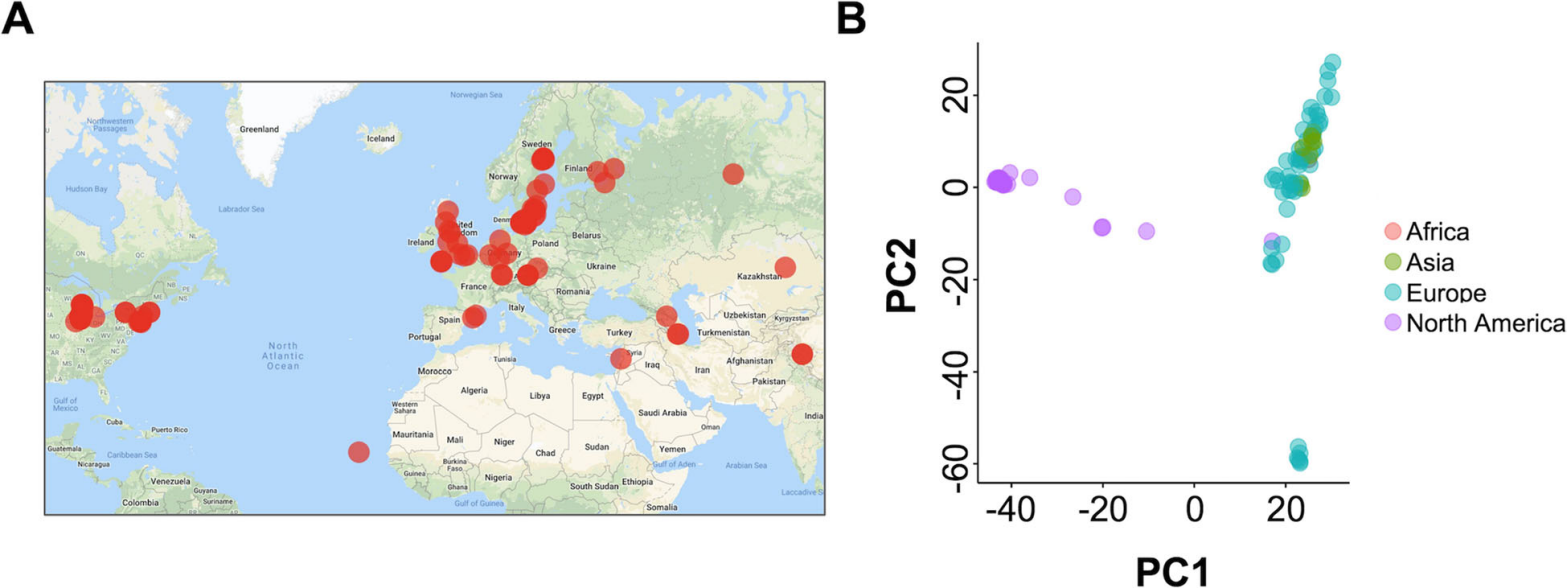
The *Arabidopsis* pan-genome. **a** Map of the 103 *Arabidopsis* accessions that were assembled in this study. **b** Principal components analysis of the structural variant presence/absence matrix of the 103 *Arabidopsis* accessions

Given the speed of RaGOO, we sought to test its scalability by performing a pan-SV genome analysis on a large population of diverse individuals. To acquire such population-scale data, we examined the sequencing data from the 1001 Genomes Project database, which includes raw short-read sequencing data and small variant calls for 1135 *Arabidopsis thaliana* accessions [42]. We mined the 1001 Genomes Project database for sequencing data amenable to genome assembly with sufficiently deep coverage of paired-end reads (the “Methods” section). This identified 103 short-read datasets representing a wide range of accessions sampled across 4 continents (Fig. 6a). We then established draft de novo assemblies for each accession using SPAdes [43]. Finally, RaGOO utilized the TAIR 10 reference genome to create 103 chromosome-scale assemblies and associated SV calls [44]. Between 85.8 and 98.7% (mean = 96.7%) of sequence was localized into chromosomes per accession, showing that the majority of assembled sequence across the pan-genome was scaffolded into pseudomolecules, even for more divergent accessions. The structural variant calls from this pan-genome provide a database of *A. thaliana* genetic variation previously unreported in the initial 1001 Genomes Project analysis [45].

SV calls were compared with SURVIVOR, yielding a total of 137,111 merged variants across the pan-genome. From this merged set of variants, we constructed a presence/absence matrix representing which variants were present in which accessions. Principal components analysis of this matrix revealed a clustering of accessions according to their geographic location (Fig. 6b). Upon further analysis of global trends in the data, we found that SVs were concentrated in pericentromeric regions, consistent with previous findings (Additional file 1: Figure S7) [46].

We further examined those genes that intersected variants present in small and large numbers of accessions, as these represent rare variants in the population and rare variants in the reference genome, respectively. When including variants present in at least 1, 10, 50, and 100 samples, we found 26,795, 17,593, 7859, and 332 total intersecting protein-coding genes (2 kbp flanking each side), respectively. Since there are a total of 27,416 protein-coding genes in the TAIR 10 database, we conclude that SVs in the pan-genome impact the genomic architecture for the majority of protein-coding genes, though fewer genes are affected by variants present in multiple samples. The full catalog of the gene structural variations is presented in Additional file 6: Table S7, and the 10 most frequently affected genes are presented in Table 2. Interestingly, most of these highly variable genes are defense response genes. Ultimately, our analysis highlights the importance of chromosome-level assembly at a population scale to help understand the broad impact of structural variation.

**Table 2:** Summary of the ten most variable genes in the *Arabidopsis* pan-genome. “Number of variants” is the total number of variants intersecting a given gene, and “Normalized number of variants” is the number of intersecting variants divided by gene length

Gene	Annotation	Number of variants	Normalized number of variants	Number of accessions with variants
AT4G16960	Defense response, chloroplast	62	0.00715605	80
AT1G58602	ADP binding, defense response, ATP binding	57	0.00244101	90
AT3G44400	ADP binding, defense response, cytoplasm, signal transduction	56	0.00621256	89
AT3G44630	Defense response	55	0.00593312	84
AT4G16920	Defense response, chloroplast, cytoplasm	55	0.00522913	79
AT1G62620	*N*,*N*-dimethylaniline monooxygenase activity, flavin adenine dinucleotide binding, NADP binding, monooxygenase activity, nucleus, oxidation-reduction process	54	0.00850796	91
AT4G16950	Defense response to fungus, incompatible interaction, nucleotide binding, defense response, protein binding	54	0.00558486	70
AT1G62630	Defense response, ATP binding, N-terminal protein myristoylation, ADP binding, nucleus	50	0.00748391	93
AT5G41740	Nucleus, defense response, chloroplast	48	0.00565171	91
AT4G16890	Defense response, cytosol, signal transduction, defense response to bacterium, protein binding, ATP binding, defense response to bacterium, incompatible interaction, ADP binding, systemic acquired resistance, salicylic acid-mediated signaling pathway, cytoplasm, intracellular membrane-bounded organelle, nucleus, nucleotide binding, endoplasmic reticulum, response to auxin	48	0.00536373	75

## Discussion

We have introduced RaGOO in both a general and focused context for highly accurate genome scaffolding. As a general method, RaGOO may be valuable for chromosome-scale scaffolding in experimental designs where ordering and/or orienting of contigs leveraging an existing reference is available. Ordering and orienting with RaGOO may also facilitate analysis not possible with unlocalized contigs. This is exemplified by the additional sequence found through gap filling of the M82, BGV, and FLA assemblies or by the identification of structural variants spanning gaps between contigs in the *S. lycopersicum* and *Arabidopsis thaliana* pan-genomes. Additionally, our pan-genome analysis demonstrates that the speed of RaGOO offers new possibilities as to the scope and size of experiments that require reference-guided scaffolding. Furthermore, the integrated structural variant identification pipeline allows for a rapid survey of gene-related and other variants in the population. This shows that for both tomato and *Arabidopsis* pan-genomes, the majority of protein-coding genes are associated with the structural variation, highlighting the importance of population-scale assembly and structural variant discovery.

In a more focused analysis, we demonstrate that RaGOO may be a valuable component of a detailed assembly pipeline to establish new high-quality eukaryotic genomic resources. Our use of RaGOO to produce three tomato assemblies highlights a valuable means of organizing contiguous draft assemblies into pseudomolecules. This is especially useful as draft assemblies become more contiguous, and high-quality references become more common, even for non-model species.

For applications that do not have independent data such as Hi-C to validate the accuracy of RaGOO output, it can be challenging to assess the extent to which errors such as reference bias are present in pseudomolecules. However, it is possible to estimate the fidelity of newly created pseudomolecules to the reference. As we show in our *S. pennellii* analysis, the percentage of localized contigs/sequence along with the RaGOO confidence scores can be examined to help determine if scaffolding was successful. In general, if pseudomolecules pass these quality control checks, users can be more confident that RaGOO pseudomolecules are accurate and complete.

## Conclusions

Our results show that RaGOO is a fast and accurate method for organizing genome assembly contigs into pseudomolecules. They also show that with a closely related reference genome, reference-guided scaffolding may yield substantially better scaffolding results than popular reference-free methods such as scaffolding with Hi-C data. In the process, we produced three tomato genome assemblies that are a valuable resource for the *Solanaceae* community and were selected to serve as the foundation for many additional tomato accessions we will be sequencing to establish a pan-SV genome for use in biology and agriculture. For this purpose, the M82 assembly has already undergone extensive procedures to provide a complete and accurate assembly with an associated set of gene models and annotations.

## Methods

### Description of RaGOO algorithm and scoring metrics

The complete RaGOO source code and documentation are available on GitHub at https://github.com/malonge/RaGOO and is released under an MIT license. RaGOO is written in Python3 and uses the python packages intervaltree and numpy. It also relies on Minimap2 that is available on GitHub at https://github.com/lh3/minimap2. RaGOO also comes bundled with an implementation of Assemblytics for structural variation analysis.

#### Scaffolding algorithm overview

RaGOO utilizes alignments to a reference genome to cluster, order, and orient contigs to form pseudomolecules. RaGOO internally invokes Minimap2, with *k*-mer size and window size both set to 19 bp, to obtain the necessary mappings of contigs to a reference genome. By default, any alignments less than 1 kbp in length are removed. To cluster contigs into chromosome groups, each contig is assigned to the reference chromosome which it covers the most. Coverage here is defined as the total number of reference chromosome base pairs covered in at least one alignment. Next, for each pseudomolecule group, the contigs in that group are ordered and oriented relative to each other. To do this, the longest (primary) alignment for each contig to its assigned reference chromosome is examined. Ordering is achieved by sorting these primary alignments by the start then end alignment position in the reference. Finally, the orientation of that contig is assigned the orientation of its primary alignment. To produce pseudomolecules, ordered and oriented contigs are concatenated, with padding of “N” characters placed between contigs.

#### Scaffolding confidence scores

Each contig is assigned a confidence score, between 0 and 1, for each of the three stages outlined above. The *clustering confidence score* is the number of base pairs a contig covered in its assigned reference chromosome divided by the total number of covered base pairs in the entire reference genome. To create a metric associated with contig ordering confidence, we defined a *location confidence*. First, the smallest and largest alignment positions, with respect to the reference, between a contig and its assigned reference chromosome are found. The location confidence is then calculated as the number of covered base pairs in this range divided by the total number of base pairs in the range. Finally, to calculate the *orientation confidence*, each base pair in each alignment between a contig and its assigned reference chromosome casts a vote for the orientation of its alignment. The orientation confidence is the number of votes for the assigned orientation of the contig divided by the total number of votes.

#### Chimeric contig correction

Prior to clustering, ordering, and orienting, RaGOO provides the option to break contigs which may be chimeric as indicated by discordant alignments to the reference. RaGOO can identify and correct both interchromosomal and intrachromosomal chimeric contigs. Interchromosomal chimeric contigs are contigs which have significant alignments to two distinct reference chromosomes. To identify and break such contigs, all the alignments for a contig are considered. Alignments less than 10 kbp are removed, and the remaining alignments are unique anchor filtered [25]. If there are multiple instances where at least 5% of the total alignment lengths cover at least 100 kbp of a distinct reference chromosome, a contig is deemed chimeric. To break the contig, alignments are sorted with respect to the contig start, then end positions, and the contig is broken where the sorted alignments transition between reference chromosomes.

Intrachromosomal chimeric contigs are contigs which have significant alignments to distant loci on the same reference chromosome. As with interchromosomal chimeric contigs, identification and breaking of intrachromosomal chimeric contigs start with removing short and non-unique alignments. The remaining alignments are sorted with respect to the start then end position in the reference chromosome. Next, the genomic distance between consecutive alignments is calculated, both with respect to the reference and the contig. If any of these distances exceeds user-defined thresholds, the contig is broken between the two alignments which the large distance between them. Only one intrachromosomal and one interchromosomal break can occur per contig per execution of the software. Importantly, all of the above criteria for breaking contigs are tunable parameters in the RaGOO software. This allows users to specify how large a structural difference between the assembly and the reference must be in order to consider it an error. Chimeric contig correction should only be used in cases when the user is confident that such large structural differences between the assembly and the reference are more likely to be misassemblies than true, large-scale structural variants. We advise users to validate misassembly correction with independent data to help ensure that true variation is not being masked.

### Scaffolding of an *Arabidopsis thaliana* and human genome

Of our 103 *A. thaliana* assemblies, we highlighted the runtime and scaffolding accuracy of the assembly representing the TFÄ 04 accession (SRR1945711). This assembly was assembled with SPAdes (see below) and had a scaffold N50 of 120,255 bp with a total size of 115,803,138 bp [43]. Additionally, to demonstrate the scaffolding of a mammalian-sized genome, we used RaGOO to order and orient the mixed haplotype human Canu assembly derived from Pacific Biosciences CCS reads. This human assembly had a contig N50 of 22,778,121 bp and a total size of 3,418,171,375 bp. For both the TFÄ 04 and human assemblies, default RaGOO parameters were used and the software was run with 8 threads (“-t 8”). The TAIR 10 and hs37d5 reference genomes were used to scaffold the TFÄ 04 and human assemblies, respectively. RaGOO completed in 12.576 s and 12 min and 33.090 s for TFÄ 04 and human, respectively. The dotplots for both assemblies were made by aligning RaGOO pseudomolecules to the respective reference genomes with nucmer (-l 200 -c 500). Alignments were filtered with delta filter (-1 -l 20000), and plots were made with Mummerplot (--fat). Only nuclear chromosome and non-alternate sequences are shown in the dotplots.

### Simulated reference-guided scaffolding

A simulated *S. lycopersicum* draft genome assembly was created by partitioning the Heinz SL3.0 reference genome, excluding chromosome 0, into scaffolds of variable length. Intervals along each chromosome were successively defined, with each interval length being randomly drawn from the distribution of observed M82 Canu contig lengths. Bedtools [47] was then used to retrieve the sequence associated with these intervals. Finally, simulated scaffolds with more than 50% “N” characters were removed, and half of the remaining contigs were randomly reverse complemented. A second simulated assembly containing contigs, rather than scaffolds, was derived from these simulated scaffolds. Scaffolds were broken at any stretch of “N” characters longer than or equal to 20 bp, excluding the gap sequence. Any resulting contigs less than 10 kbp in length were also excluded. We call this pair of simulated assemblies the “easy” set of simulated data. To simulate a “hard” set of data, we started with the same “easy” scaffolds and added variation. To do this, we used SURVIVOR to simulate 10,000 indels ranging from 20 bp to 10 kbp in size. We also added SNPs at a rate of 1%. Again, we split these scaffolds into contigs resulting in a pair of “hard” simulated assemblies.

Given these “easy” and “hard” simulated scaffolds and contigs, RaGOO, Chromosomer, and MUMmer’s “show-tiling” utility were used for reference-guided scaffolding. For RaGOO, chimera breaking was turned off, and default parameters were used with the exception of the padding amount, which was set to zero. Chromosomer utilized Blast alignments with default parameters. Additionally, the “fragmentmap ratio” was set to 1.05, and the padding amount was set to zero. Show-tiling used default parameters. Since RaGOO and Chromosomer rely on aligners that allow for multithreading, both tools were run with eight threads, while show-tiling was run with a single thread.

We recorded various measurements to evaluate the success of these tools in ordering and orienting simulated assemblies. Firstly, we observed the runtime, percentage of localized contigs, and percentage of localized sequence. To assess the clustering and orienting accuracy, we measure the percentage of localized contigs that had been assigned the correct cluster and orientation, respectively. Finally, we used two measurements to assess the ordering accuracy of each pseudomolecule. The first was the edit distance between the true and predicted order of contigs. This edit distance was normalized by dividing by the total number of contigs in the true ordering. The second ordering accuracy measurement was the percentage of correct adjacent contig pairs.

### Tomato sequencing data

#### Plant material and growth conditions

Seeds of the *S. lycopersicum* cultivar M82 (LA3475) were from our own stocks. Seeds of the *S. pimpinellifolium* accession BGV006775 were provided by E. van der Knaap, University of Georgia. Seeds of the *S. lycopersicum* breeding line Fla.8924 were from the stocks of S. Hutton, University of Florida. Seeds were directly sown and germinated in the soil in 96-cell plastic flats and grown under long-day conditions (16-h light/8-h dark) for 21 days in a greenhouse under natural light supplemented with artificial light from high-pressure sodium bulbs (~ 250 μmol m^2^ s^1^). Daytime and nighttime temperatures were 26–28 **°**C and 18–20 **°**C, respectively, with a relative humidity of 40–60%.

#### Genome and transcriptome sequences

Genomic Illumina read data for BGV006775 were downloaded from the NCBI Sequence Read Archive (SRA) database (accession SRS3394566). Genomic Illumina read data for Fla.8924 (Lee et al. [33]) was provided by S. Hutton, University of Florida. Illumina read data for all transcriptomes were downloaded from ftp://ftp.solgenomics.net/user_requests/LippmanZ/public_releases/by_experiment/Park_etal/ [SeSo1] ftp://ftp.solgenomics.net/transcript_sequences/by_species/Solanum_lycopersicum/libraries/illumina/LippmanZ/; [SeSo2] http://solgenomics.net/[SeSo3]. [SeSo4] [ZBL5].

#### Tissue collection and high molecular weight DNA extraction

For extraction of high molecular weight DNA, young leaves were collected from 21-day-old light-grown seedlings. Prior to tissue collection, seedlings were incubated in complete darkness for 48 h. Flash-frozen plant tissue was ground using a mortar and pestle and extracted in five volumes of ice-cold extraction buffer 1 (0.4 M sucrose, 10 mM Tris-HCl pH 8, 10 mM MgCl_2_, and 5 mM 2-mercaptoethanol). Extracts were briefly vortexed, incubated on ice for 15 min, and filtered twice through a single layer of Miracloth (Millipore Sigma). Filtrates were centrifuged at 4000 rpm for 20 min at 4 **°**C, and pellets were gently re-suspended in 1 ml of extraction buffer 2 (0.25 M sucrose, 10 mM Tris-HCl pH 8, 10 mM MgCl_2_, 1% Triton X-100, and 5 mM 2-mercaptoetanol). Crude nuclear pellets were collected by centrifugation at 12,000*g* for 10 min at 4 **°**C and washed by re-suspension in 1 ml of extraction buffer 2 followed by centrifugation at 12,000*g* for 10 min at 4 **°**C. Nuclear pellets were re-suspended in 500 μl of extraction buffer 3 (1.7 M sucrose, 10 mM Tris-HCl pH 8, 0.15% Triton X-100, 2 mM MgCl_2_, and 5 mM 2-mercaptoethanol), layered over 500 μl extraction buffer 3, and centrifuged for 30 min at 16,000*g* at 4 **°**C. The nuclei were re-suspended in 2.5 ml of nuclei lysis buffer (0.2 M Tris pH 7.5, 2 M NaCl, 50 mM EDTA, and 55 mM CTAB) and 1 ml of 5% Sarkosyl solution and incubated at 60 **°**C for 30 min. To extract DNA, nuclear extracts were gently mixed with 8.5 ml of chloroform/isoamyl alcohol solution (24:1) and slowly rotated for 15 min. After centrifugation at 4000 rpm for 20 min, ~ 3 ml of aqueous phase was transferred to new tubes and mixed with 300 μl of 3 M NaOAC and 6.6 ml of ice-cold ethanol. Precipitated DNA strands were transferred to new 1.5 ml tubes and washed twice with ice-cold 80% ethanol. Dried DNA strands were dissolved in 100 μl of elution buffer (10 mM Tris-HCl, pH 8.5) overnight at 4 **°**C. Quality, quantity, and molecular size of DNA samples were assessed using Nanodrop (Thermofisher), Qbit (Thermofisher), and pulsed-field gel electrophoresis (CHEF Mapper XA System, Biorad) according to the manufacturer’s instructions.

#### Nanopore library preparation and sequencing

DNA was sheared to 30 kb using the Megarupter or 20 kb using Covaris g-tubes. DNA repair and end-prep was performed using New England Biosciences kits NEBNext FFPE DNA Repair Kit and Ultra II End-Prep Kit. DNA was purified with a 1× AMPure XP bead cleanup. Next, DNA ligation was performed with NEBNext Quick T4 DNA Ligase, followed by another AMPure XP bead cleanup. DNA was re-suspended in elution buffer and sequenced according to the MinION standard protocol.

#### 10× Genomics library preparation and sequencing

1.12 ng of high molecular weight gDNA was used as input to the 10× Genomics Chromium Genome kit v2 and libraries we prepared according to the manufacturer’s instructions. The final libraries, after shearing and adapter ligation, had an average fragment size of 626 bp and were sequenced on an Illumina HiSeq, 2500 2 × 250 bp.

#### Hi-C library preparation and sequencing

DNA extraction, library construction, and sequencing for Hi-C analyses was performed by Phase Genomics (Seattle, WA) and conducted according to the supplier’s protocols. Young leaves from 21-day-old light-grown and 48-h dark-incubated seedlings were wrapped in wet tissue paper and shipped on ice overnight.

### Initial de novo assembly of tomato genomes

The Oxford Nanopore sequencing data for M82, BGV, and FLA were assembled with Canu. For all three assemblies, default parameters were used with the expected genome size set to 950 Mbp. Assemblies were submitted to the UGE cluster at Cold Spring Harbor Laboratory for parallel computing. After assembly, it was determined that the M82 assembly contained bacterial contamination. To remove bacterial contigs from the assembly, the Canu contigs were aligned to all RefSeq bacterial genomes (downloaded on June 7, 2018) as well as the Heinz SL3.0 reference genome. If a contig covered more RefSeq bacterial genome base pairs than SL3.0 base pairs, it was deemed a contaminant and removed from the assembly. In this paper, “M82 Canu contigs” refers to the Canu contigs after contaminant contigs had been removed.

### Reference-guided and reference-free scaffolding of tomato genomes

The M82 Canu contigs were ordered and oriented into pseudomolecules with RaGOO, Chromosomer, and Nucmer’s “show-tiling” utility. The Heinz SL3.0 reference, with chromosome 0 removed, was used for all tools. RaGOO used eight threads with chimeric contig correction turned on and the gap padding size set to 200 bp. We also instructed RaGOO to skip three contigs which had low grouping accuracy scores. Chromosomer used eight threads for BLAST alignments. The Chromosomer fragmentmap ratio was set to 1.05, and the gap padding size was set to 200 bp. Default parameters were used for show-tiling.

For reference-free scaffolding of the M82 assembly, 46,239,525,282 bp (~ 60× coverage of the M82 Canu contigs) of 2 × 101 Hi-C sequencing reads were aligned to the M82 Canu contigs with BWA mem using the “-5” flag [48]. Aligned reads were then filtered with “samtools view” to include alignments where both mates of a pair aligned as a primary, non-supplementary alignments (-F 2316) [49]. SALSA2 then utilized these alignments along with the M82 Canu assembly graph to build scaffolds. The SALSA2 “-m” flag was also set to “yes” in order to correct misassemblies in the M82 contigs, and the expected genome size was set to 800 Mbp. Finally, we set “-e GATC” to correspond to the use of Sau3AI in the Hi-C library. The SALSA2 scaffolds were comprised of 2065 scaffolds and had an N50 of 18,282,950 bp and a total size of 827,545,698 bp.

The structural accuracy of the M82 RaGOO pseudomolecules and SALSA2 scaffolds was assessed with dotplots and Hi-C density plots. For dotplots, both sequences were aligned to the Heinz SL3.0 reference (with chromosome 0 removed) with Minimap2 using the “-ax asm5” parameter. Alignments less than 12 kbp in length were excluded. For Hi-C visualization, the same Hi-C data described earlier was aligned to both sequences using the same parameters as were used for SALSA2. These alignments were then visualized with Juicebox [50]. Hi-C mates that mapped to the same restriction fragment were excluded from visualization.

Using the same parameters as M82, RaGOO was also used to order and orient the FLA and BGV Canu assemblies. BGV underwent two rounds of chimeric contig correction. Assemblytics structural variants for each assembly were compared with “SURVIVOR merge,” with the “max distance between breakpoints” set to 1 kbp. Variants in chromosome 0 of the SL3.0 reference as well as variants which spanned more than 10% gaps were excluded from the structural variant analysis.

### Tomato genome correction and polishing

M82 RaGOO pseudomolecules were manually corrected for misassemblies and/or reference bias. Manual corrections were identified by visualizing Hi-C alignments to the M82 genome described in the previous sections. Firstly, three contigs with spurious alignments were removed from the pseudomolecules. Then, using Juicebox Assembly Tools, an inversion error was corrected on chromosome 3 and two ordering errors were corrected, one on chromosome 7 and one on chromosome 11. The “.assembly” file associated with these manual edits can be found in Additional file 7. Gap filling and polishing was performed on the RaGOO pseudomolecules for the M82, FLA, and BGV tomato accessions. For each assembly, all respective Oxford Nanopore sequencing data used for assembly was used for gap filling with PBJelly.

After gap filling, we sought to find the most effective genome polishing strategy given our data. We used the gap-filled M82 assembly as a starting point for our tests. To polish this genome, we utilized the raw Oxford Nanopore data used for assembly as well as 10× Genomics Illumina Whole Genome Shotgun sequencing reads. We trimmed adapters and primers (23 bp from the beginning of read 1) and low-quality bases (40 bp from the ends of read 1 and read 2) from these 10× genomics data. With these data, we compared multiple polishing strategies using various alignment and polishing tools. First, we examined assemblies polished with or without Nanopolish [51]. For Nanopolish, the M82 raw Oxford Nanopore read set was aligned to the M82 assembly with Minimap2 using the “map-ont” parameter. Next, we compared assemblies polished with 1 or 2 rounds of Pilon polishing. For each round of polishing, the Illumina data was randomly subsampled to 40× coverage prior to alignment. Finally, we compared bwa mem, Bowtie2, and ngm for short-read alignment prior to Pilon polishing [52, 53]. We used bwa mem and ngm with default parameters, while Bowtie2 was run with the “--local” parameter.

We used MUMmer’s “dnadiff” utility to compare the efficacy of these polishing pipelines (Additional file 3). For dnadiff analysis, polished assemblies and the SL3.0 reference were broken into contigs by breaking sequences at gaps of 20 bp or longer. Then, assemblies were aligned to the reference contigs with nucmer using the “-l 100 -c 500 –maxmatch” parameters. After determining that 2 rounds of Pilon polishing with Bowtie2 yielded the best results, we applied the same pipeline to the BGV and FLA assemblies using ~ 23× coverage and ~ 26× coverage of paired-end Illumina short-read data was used for BGV and FLA, respectively. BUSCO was used to evaluate genome completeness of the polished M82, BGV, and FLA assemblies. The Solanaceae odb10 database was used with the “species” parameter set to “tomato.”

Finally, we searched for spurious duplications introduced after gap-filling with PBJelly, since others have reported such phenomena [54]. We first examined the M82, BGV, and FLA assemblies after gap-filling but before polishing. Using these assemblies, we called structural variants with respect to the SL3.0 reference genome using Assemblytics (unique minimum alignment length set to 10 kbp). We then found all “tandem expansions” (duplications) that intersected gaps filled by PBJelly. Finally, for any intersecting tandem expansions, we calculated the average raw ONT read coverage across the variant. For FLA and BGV, all tandem expansions in filled-gaps had ample read support (> 15×). For M82, there were two tandem expansions that had less than 1× coverage. Since one variant was only 7 bp long with respect to the M82 assembly, we omitted it from this analysis. The remaining spurious tandem expansion extended 982 bp and was perfectly mapped to the final polished M82 assembly using Minimap2 to M821.3ch09: 21470172-21471154.

### Tomato genome annotation

We annotated protein-coding genes in the M82, FLA, and BGV assembly using the Maker v3.0 pipeline on Jetstream by providing repeats, full length cDNA sequences, and proteins from Heinz 1706 ITAG3.2 assembly [55]. Simple, low-complexity, and unclassified repeats were excluded from masking. We additionally provided Maker with an M82 reference transcriptome derived from 50 M82 RNA-seq libraries. RNA-seq reads were aligned to the M82 genome using STAR, a splice aware aligner [56]. These alignments were used to assemble transcripts and establish a consensus transcriptome using StringTie and TACO, respectively [57, 58]. We ran Maker using parameters est2genome set to 1, protein2genome set to 1 and keep_preds set to 1 to perform the gene annotation. Low consensus gene models with an AED score above 0.5 were filtered from the Maker-predicted gene models. We additionally removed gene models shorter than 62 bp following the cutoffs used for the ITAG3.2 annotation. Putative gene functions were assigned to the MAKER gene models via Interproscan protein signatures and blastp protein homology search [18]. blastp queried the UniProtKB/Swiss-Prot and Heinz 1706 ITAG3.2 protein databases, filtering out alignments with an *e* value greater than 1e−05 [59]. We further filtered out genes that did not have an associated gene function in either Interproscan, UniprotKB/Swiss-Prot, or ITAG3.2.

#### *S. pennellii* genome scaffolding

*S. pennellii* contigs were scaffolded with both the Heinz 1706 SL3.0 reference and the independent *S. pennellii* reference genome using default RaGOO parameters and excluding chromosome 0 from the reference chromosomes (“-e”). The two resulting sets of pseudomolecules were aligned to each other using Nucmer (-l 200 -c 500). The resulting alignments were filtered with delta-filter (-l 50000 -1) and plotted with mummerplot. The two reference genomes were also aligned to each other using Nucmer (-l 50 -c 100), and the resulting alignments were filtered with delta-filter (-l 10000 -1) and plotted with mummerplot.

### Arabidopsis structural variant analysis

The 1001 Genomes Database was mined for accessions for which there was at least 50× coverage of paired-end sequencing data. We also required that the read length be at least 100 bp. For practical reasons, we excluded accessions with excessive coverage. For each of the remaining accessions, the fastq files were randomly subsampled in order to achieve exactly 50× coverage. Subsampled reads were then assembled with the SPAdes assembler, with *k*-mer size set to 33, 55, 77, and 99, otherwise default parameters. These draft assemblies were then ordered and oriented with RaGOO using default parameters (no chimeric contig correction) and the TAIR 10 reference genome (GCA_000001735.1). RaGOO also provided structural variants, with the minimum variant size set to 20 bp. Of the chromosome-scale assemblies, a few assemblies with a genome size greater than 150 Mbp were removed due to putative sample contamination. After this filtering, assemblies and structural variant calls for 103 accessions remained.

Variants that were called in chromosome 0 or the chloroplast/mitochondrial chromosomes were discarded. Also, variants which had more than a 10% overlap with a gap were excluded. To find unique variants across multiple samples, SURVIVOR merge was used such that a variant only had to be present in at least 1 sample for it to be reported. Therefore, given all 103 samples, this yielded the union of all variants present in the pan-genome. To find shared variants across multiple samples, SURVIVOR merge was used such that a variant must have been present in all samples to be reported. This effectively provided the intersection of variants in the pan-genome. In all instances of using SURVIVOR merge, the “max distance between breakpoints” was set to 1 kbp. Also, the strand of the SV was taken into account, while distance based on the size of the variant was not estimated. Finally, the minimum variant size was set to 20 bp to be consistent with the RaGOO parameters. Bedtools was used to find variant/gene intersections.

## Supplementary information

**Additional file 1: Figure S1.** Arabidopsis and human assembly dotplots. **Figure S2.** M82 RaGOO confidence score distribution. **Figure S3.** Heinz cDNA alignment. **Figure S4.** Annotation Edit Distances. **Figure S5.** *S. pennellii* dotplots. **Figure S6.** *S. pennellii* confidence score distributions. **Figure S7.** *A. thaliana* pan-genome SV distribution. **Table S1.** Sequence statistics for simulated tomato genomes. **Table S3.** Performance statistics for Tomato chromosome construction.

**Additional file 2: Table S2.** Chromosome construction accuracy and runtime statistics.

**Additional file 3: Table S4.** Accuracy of different polishing strategies of M82.

**Additional file 4: Table S5.** Maker gene annotation statistics.

**Additional file 5: Table S6.** Variants intersecting tomato genes across the Pan-Genome.

**Additional file 6: Table S7.** Variants in Arabidopsis Genes across the Pan-Genome.

**Additional file 7:** “.assembly” file for JuiceBox Assembly Tools M82 analysis.

**Additional file 8:** The review history.

## Data Availability

RaGOO is available open source under the MIT license at https://github.com/malonge/RaGOO [26]. The tomato genomes are also available in the Sol Genomics Network (ftp://ftp.solgenomics.net/genomes/Solanum_lycopersicum/M82/, ftp://ftp.solgenomics.net/genomes/Solanum_lycopersicum/Fla.8924/, ftp://ftp.solgenomics.net/genomes/Solanum_pimpinellifolium/BGV006775/), and the raw sequencing data are available in the Sequence Read Archive under BioProject PRJNA557253. Sequencing data, genome assemblies, annotations, and structural variation calls for all samples are available at http://share.schatz-lab.org/ragoo/.
