# Supplementary material for: RaGOO: fast and accurate reference-guided scaffolding of draft genomes
Source: Genome Biol. 2019 Oct 28;20:224. doi: 10.1186/s13059-019-1829-6 (PMC6816165; doi:10.1186/s13059-019-1829-6)
Supplement: Supplementary file 1 — Additional file 1: Figure S1. Arabidopsis and human assembly dotplots. Figure S2. M82 RaGOO confidence score distribution. Figure S3. Heinz cDNA alignment. Figure S4. Annotation Edit Distances. Figure S5. S. pennellii dotplots. Figure S6. S. pennellii confidence score distributions. Figure S7. A. thaliana pan-genome SV distribution. Table S1. Sequence statistics for simulated tomato genomes. Table S3. Performance statistics for Tomato chromosome construction. [file 13059_2019_1829_MOESM1_ESM.docx]

**RaGOO: Fast and accurate reference-guided scaffolding of draft genomes**

Michael Alonge^1^, Sebastian Soyk^2^, Srividya Ramakrishnan^1^, Xingang Wang^2^, Sara Goodwin^2^, Fritz J. Sedlazeck^3^, Zachary B Lippman^2,4^, Michael C. Schatz^1,2,5^

**Supplementary Materials**

[Fig S1. Arabidopsis and Human Assembly Dotplots. 2](#_Toc18349304)

[Fig S2. M82 RaGOO Confidence Score Distribution. 3](#_Toc18349305)

[Fig S3. Heinz cDNA Alignment. 4](#_Toc18349306)

[Fig S4. Annotation Edit Distances. 5](#_Toc18349307)

[Fig S5. *S. pennellii* Dotplots. 6](#_Toc18349308)

[Fig S6. *S. pennellii* Confidence Score Distributions. 7](#_Toc18349309)

[Fig S7. *A. thaliana* Pan-Genome SV Distribution. 8](#_Toc18349310)

[Table S1. Sequence statistics for simulated tomato genomes 9](#_Toc18349311)

[Table S2. Performance statistics for Tomato chromosome construction. 9](#_Toc18349312)


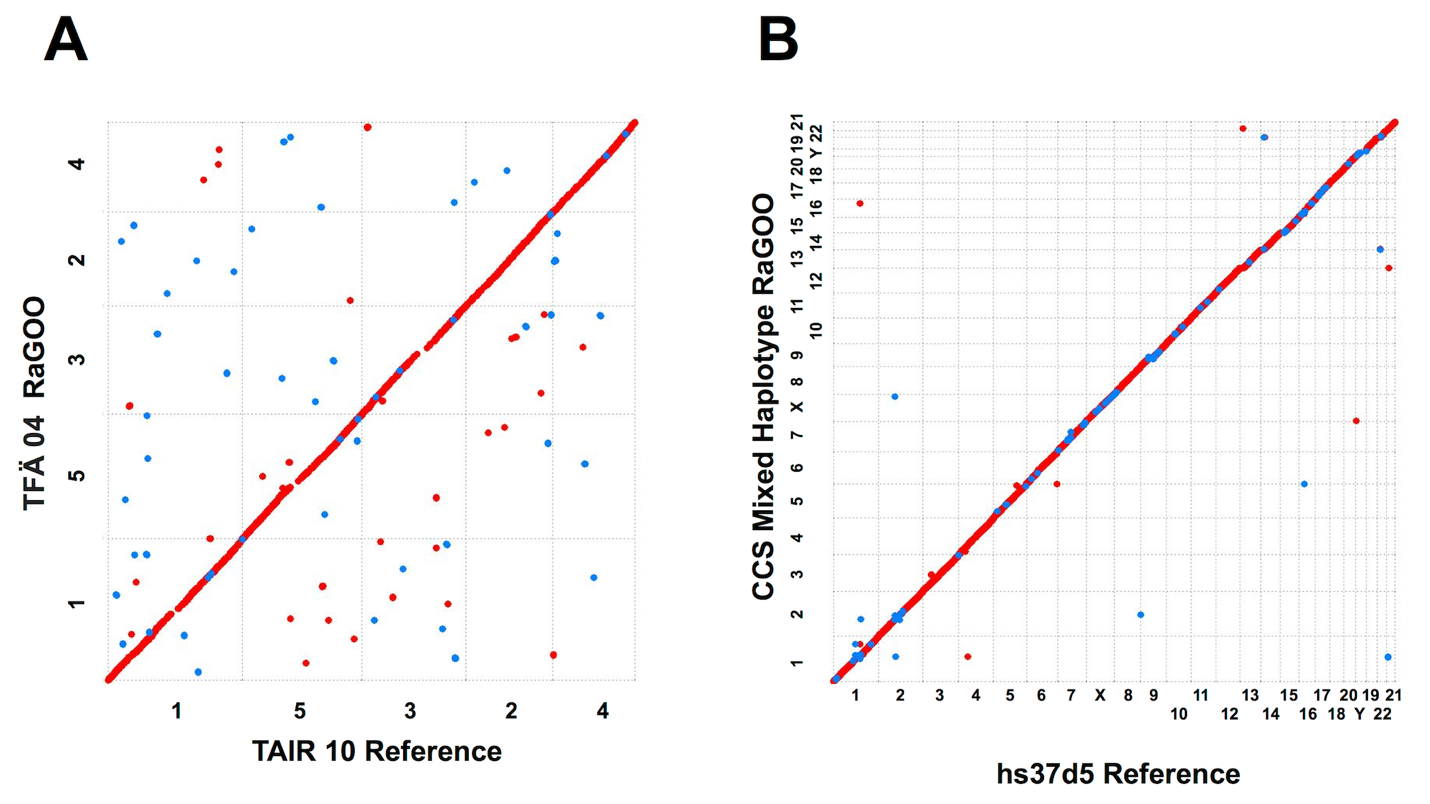


Fig S1. Arabidopsis and Human Assembly Dotplots. Dotplots depicting alignments between **(A)** TFÄ 04 RaGOO pseudomolecules and the TAIR 10 reference and **(B)** CCS mixed haplotype RaGOO pseudomolecules and the hs37d5 reference.


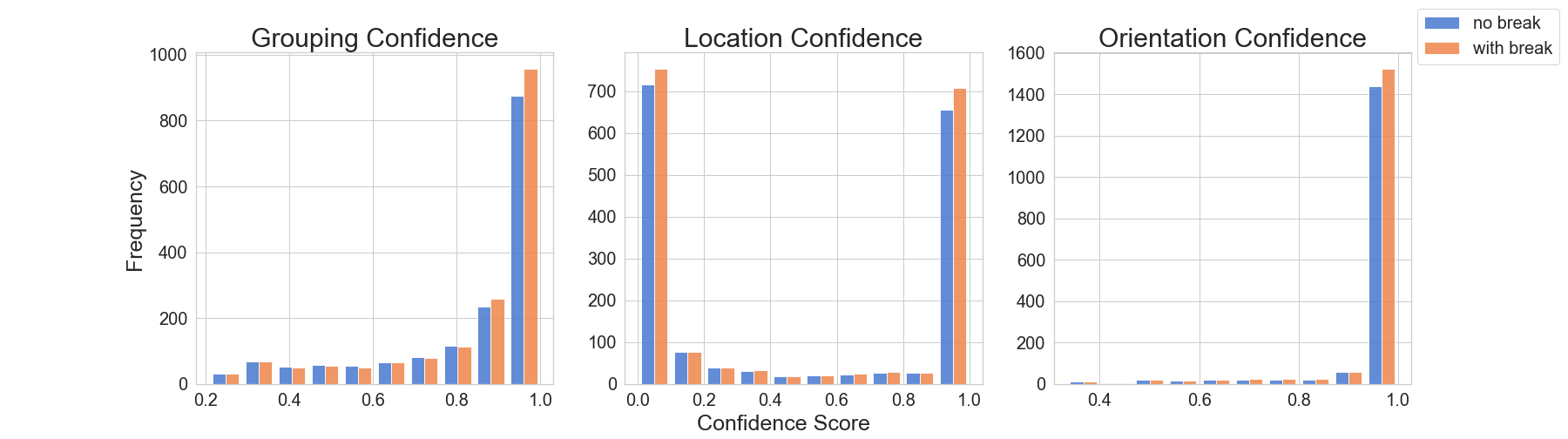


Fig S2. M82 RaGOO Confidence Score Distribution. RaGOO grouping, location and orientation confidence score distributions for M82 scaffolding, both with and without chimeric contig correction (“with break” and “no break” respectively).


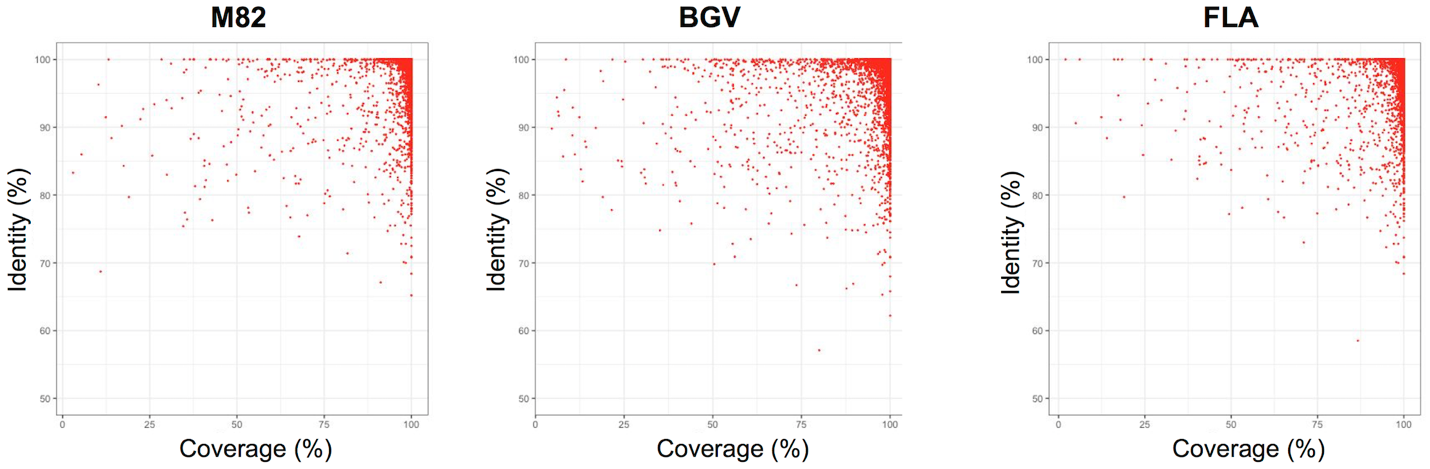


Fig S3. Heinz cDNA Alignment. Coverage vs. identity of GMAP alignments of ITAG3.2 cDNA to the M82, BGV, and FLA assemblies.


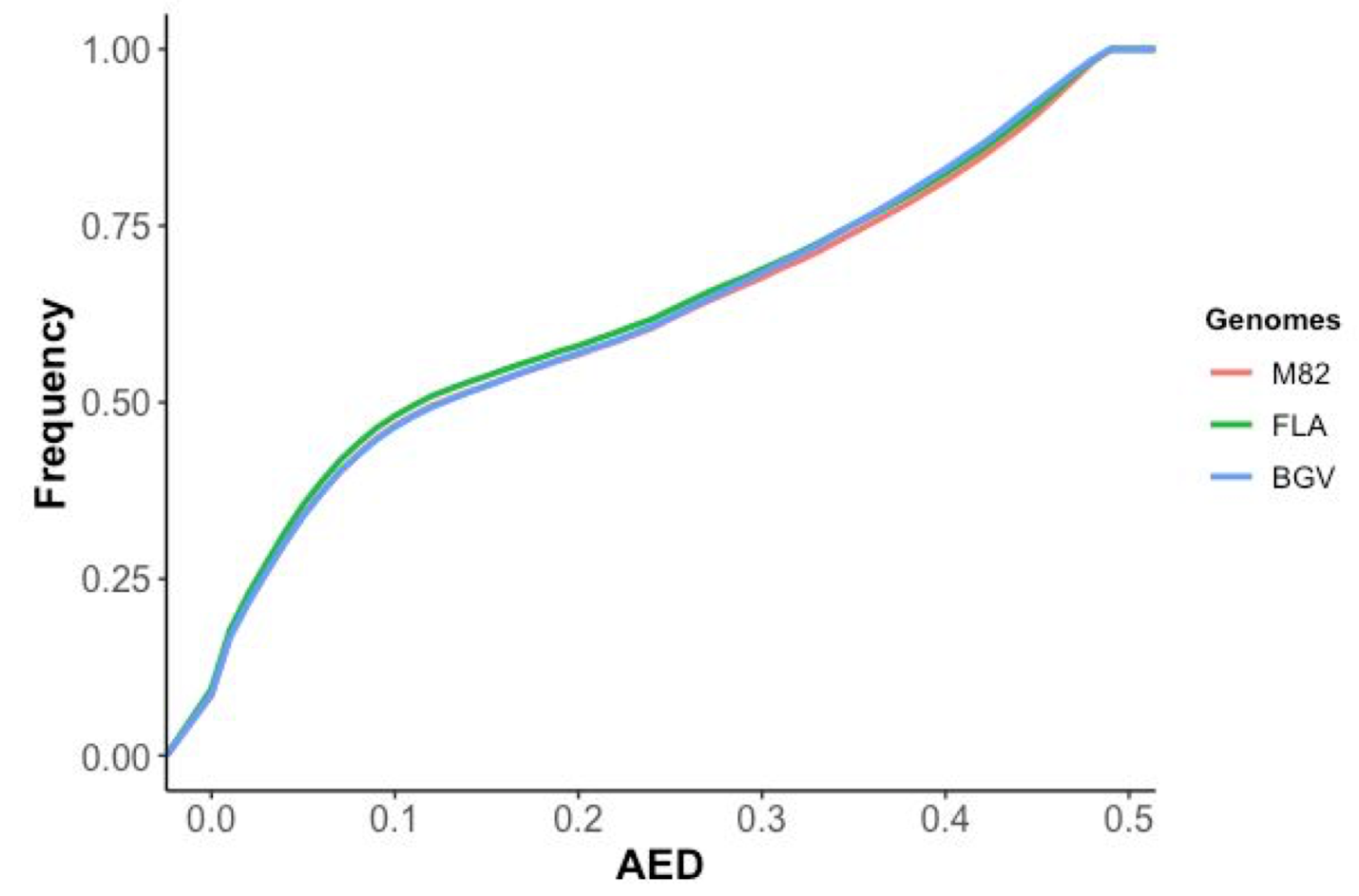


Fig S4. Annotation Edit Distances. Cumulative Distribution of Annotation Edit Distance (AED) in Maker annotated Genomes.


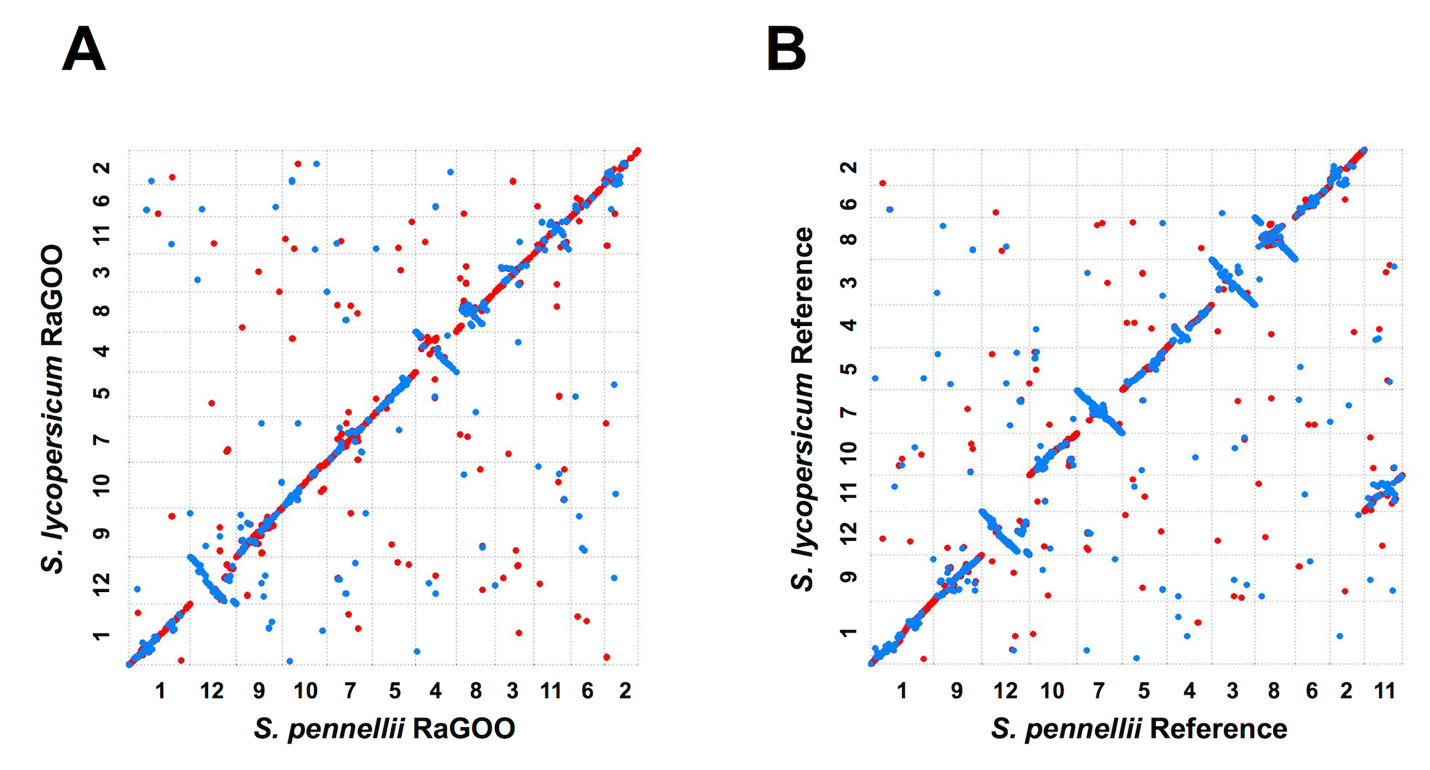


Fig S5. *S. pennellii* Dotplots. **(A)** Dotplot depicting alignments between two sets of pseudomolecules derived from the same set of S. pennellii contigs. The x-axis represents pseudomolecules established according to an independent S. pennellii reference genome, while the y-axis represents pseudomolecules established according to the SL3.0 S. lycopersicum reference genome. **(B)** Dotplot depicting the two aforementioned reference genomes aligned to each other.


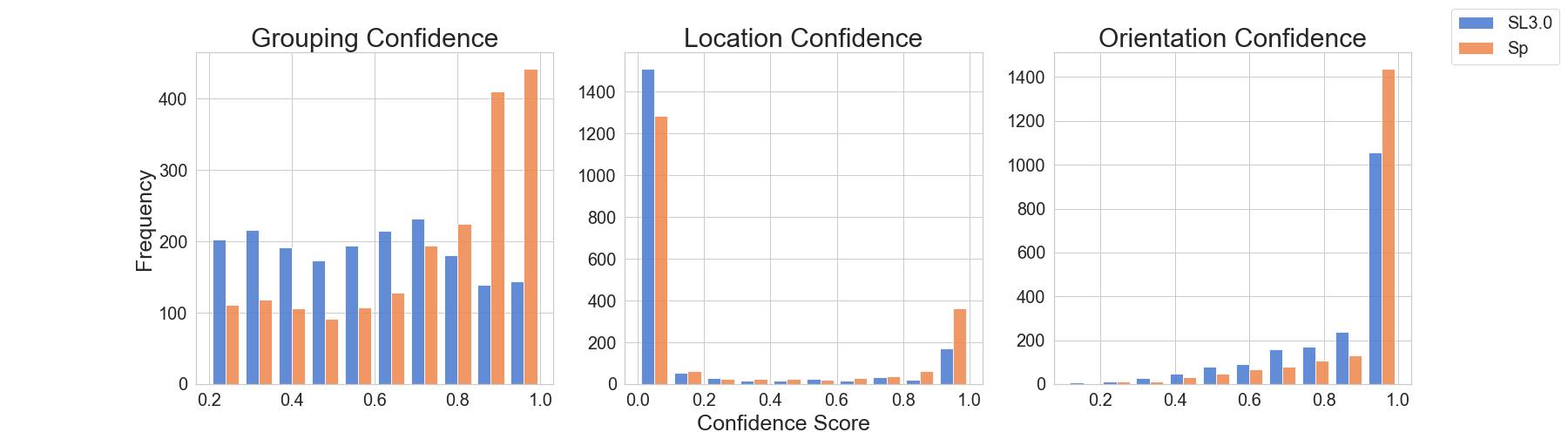


Fig S6. *S. pennellii* Confidence Score Distributions. RaGOO grouping, location and orientation confidence score distributions of *S. pennellii* contigs when using the *S. lycopersicum* reference (“SL3.0”) and when using the independent *S. pennellii* reference (“Sp”).


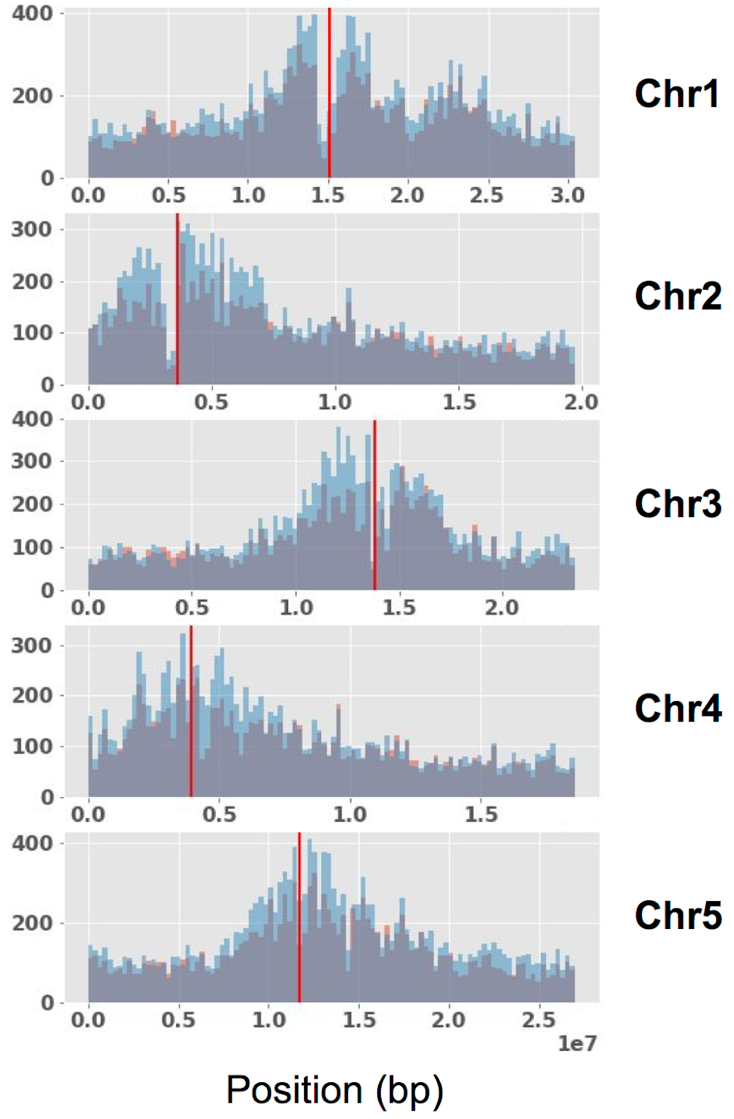


Fig S7. *A. thaliana* Pan-Genome SV Distribution. Distribution of insertions (orange) and deletions (blue) along each of the 5 *Arabidopsis thaliana* chromosomes. The red vertical lines indicate the centromere midpoints.

## Table S1. Sequence statistics for simulated tomato genomes

| Assembly | # scaffolds/contigs | N50 (bp) | Total Sequence (bp) |
| --- | --- | --- | --- |
| Easy Scaffolds | 1,751 | 1,579,621 | 776,505,792 |
| Easy Contigs | 10,593 | 123,451 | 685,720,624 |
| Hard Scaffolds | 1,604 | 1,587,209 | 776,241,593 |
| Hard Contigs | 10,456 | 124,354 | 685,812,284 |

## Table S3. Performance statistics for Tomato chromosome construction.

| Tool | % Genome Localized | % Contigs Localized | Run Time | # Cores |
| --- | --- | --- | --- | --- |
| show-tiling | 3.17 | 13.81 | 285m 6.652s | 1 |
| Chromosomer | 85.6 | 83.67 | 1466m 41.274s | 8 |
| RaGOO | 99.01 | 95.62 | 7m 15.123s | 8 |
