# Supplementary material for: RaGOO: fast and accurate reference-guided scaffolding of draft genomes
Source: Genome Biol. 2019 Oct 28;20:224. doi: 10.1186/s13059-019-1829-6 (PMC6816165; doi:10.1186/s13059-019-1829-6)
Supplement: Supplementary file 8 — Additional file 8: The review history. [file 13059_2019_1829_MOESM8_ESM.docx]

**Review history**

**First round**

**Reviewer 1**

I read the manuscript by Alonge M. et al entitled "Fast and accurate reference-guided scaffolding of draft genomes" with great interest. The authors describe their algorithm dedicated to the scaffolding of draft assemblies using existing reference genomes.
The RaGOO software is available online, easy to install and use. I tested RaGOO on my own dataset and it allowed me to get chromosome-scale assemblies in a very short time compared to existing tools. There is no real novelty because the gain is due to the fast aligner minimap2; however the RaGOO software is a well-integrated method that allows large-scale genomic projects to obtain chromosome-scale assemblies at a lower cost. Given these elements, I think this deserves a publication.

Major points:

- The authors listed existing tools for scaffolding from a given reference genome, but RACA was not mentioned. In addition, the authors argue that several tools are not effective on large genomes, but RACA and Ragout2 have been used on eukaryotic genomes and they should achieve the goal. Rebaler (https://github.com/rrwick/Rebaler) is another alternative based on a reference and a long read dataset.

- ONT data as well as tomato genome assemblies and their annotation should be submitted to public databases such as NCBI or EBI.

- The algorithm is not sufficiently described in the Methods section, especially in the subsection related to the chimeric contig correction. It is unclear what are the differences between a real interchromosomal rearrangement and an interchromosomal chimeric contig, as well as between a deletion and an intrachromosomal chimeric contig. The authors should add a figure to further explain the algorithm; Figure 1 is not very useful.

- Assigning a confident score is an interesting feature. I think the authors should discuss the scoring method in the main manuscript and show on the genomes of tomato and Arabidopsis how the score can be useful. "These scores can also be used to assess the efficacy of chimeric contig correction or to check if an assembly diverges too much from the reference genome for accurate scaffolding" (P9, L42). I think the authors should show how users can evaluate the accuracy of the scaffolding using confident scores.

- I tested the RaGOO software on several assemblies. On genome assemblies <1G, it runs pretty fast and the ordering of contigs is reliable. However I was surprised to find that it does not produce an AGP file that can be useful for transferring annotations. On a larger genome (>10 Gb), the alignment step was fast but the software spent 5 days generating pseudomolecules without providing information, which can be disconcerting for users.

- I was wondering how RaGOO handles the scaffolding with a more distant reference genome; it should increase the size of the Chr0 sequence. Another tomato genome has been sequenced using the nanopore technology, and because it is not the same species (Solanum pennellii, http://www.plantcell.org/content/29/10/2336) it should be interesting to use it for a test. 

- There are big differences in the contiguity of the three tomato genomes, can the authors explain the reason for these differences. In addition, the assembly of the GBV genome, which has the highest contiguity, contains more structural variants than M82 and FLA, do you think it could be related to the high contiguity of the initial Nanopore assembly?

- The authors chose to polish the Nanopore consensus after the scaffolding step, but error correction could improve the minimap2 alignment step. Can the authors comment?

- A gap-filling step was performed using PBJelly but a previous study found that PBJelly generates artefactual duplications (https://www.nature.com/articles/sdata2018235).  It would be good to check these artifacts as they can lead to the prediction of false structural variants.


Minor points:

- Was the chimeric contig correction turned on for the scaffolding of the Arabidopsis assemblies? I cannot find the information in the manuscript.

- The Supplementary Tables are either missing or poorly formatted in the pdf file I received.

- P2: "Acquiring a genomic map could be expensive". Two recent studies have reported chromosome-scale assemblies based on long reads and optical maps with reasonable costs (10.1038/s41477-018-0289-4 and 10.1038/s41467-018-07271-1).

- P3: "can optionally avoid breaking chimeric intervals at loci within…". I think this is an interesting feature, because it will allow the scaffolding of already annotated assemblies. 

- P5: Please provide the standard metrics of the SALSA2 assembly.

- P8: "… to uncover genomic variation not discovered through traditional short-read mapping". The authors must prove that these genomic variations could not be discovered using short reads or delete this sentence. 

- P13: "BGV underwent two rounds of chimeric contig correction.". Why is the process different for the assembly of the BGV genome?

**Reviewer 2**

The paper is devoted to a newly developed open-source tool RaGOO which is intended to accurately reconstruct 
contig ordering and orientation guided by a reference genome. RaGOO  (based on Minimap2 aligner) is very fast in comparison with available tools 
(~1B bp plant genome is processed in 7 min).

There two types of results - validation of the tool and report on newly
identified structural variations in plant genomes.

In general, I believe that application to plant genomes may be more stressed  in conclusions -
it is not clear how well it will performed on animal genomes. Specifically, the main competitor is Chromosomer
which was originally applied to insects.

RaGOO has been successfully applied to tomato genome (M82 reference cultivar).
Validation of scaffolding results using  de novo assemblies of
Oxford Nanopore long-read sequencing is a significant advantage of the study.
It allowed show that the tool is generally very accurate. 

Numerous structure variations
which were not recorded before have been identified for M82.
A pan-genome analysis of 103 Arabidopsis
thaliana accessions also identified newly detected structural variations. 


- The methods are appropriate and well described.

- Conclusions are adequately supported by results and shown data. 

- Sufficient details  are provided to allow replication and comparison with related analyses.

- The software (available at github) has been compared with existing tools (Chromosomer and show-tiling) and show advantages both in quality and efficiency. 
  It would be advantageous to apply to non-plant genomes - although i would not require that for the publication. 


The paper is of broad interest to bioinformaticians specialized in sequence assembly as well as to biologists who assemble large genomes  especially plant genomes.
The paper and the results including a new annotated release of M82 tomato genome will be of particular interest to plant biologists. The tool can be used by a very
broad audience for assembly of large genomes, finding large structural variations, and annotating pan-genomes.


Minor comments:
- mention that validation are on plant genomes.
- Supplementary Table 1 is missing?
- Supplementary Table 3 is impossible to read since it is partitioned between pages and there is no pointer to excel

**Response to reviewers**

Reviewer #1: I read the manuscript by Alonge M. et al entitled "Fast and accurate referenceguided

scaffolding of draft genomes" with great interest. The authors describe their algorithm

dedicated to the scaffolding of draft assemblies using existing reference genomes.

The RaGOO software is available online, easy to install and use. I tested RaGOO on my own

dataset and it allowed me to get chromosome-scale assemblies in a very short time compared

to existing tools. There is no real novelty because the gain is due to the fast aligner minimap2;

however the RaGOO software is a well-integrated method that allows large-scale genomic

projects to obtain chromosome-scale assemblies at a lower cost. Given these elements, I think

this deserves a publication.

Major points:

Personal Cover Click here to access/download;Personal

Cover;review_responses_resub1.pdf

- The authors listed existing tools for scaffolding from a given reference genome, but RACA was

not mentioned. In addition, the authors argue that several tools are not effective on large

genomes, but RACA and Ragout2 have been used on eukaryotic genomes and they should

achieve the goal. Rebaler (https://github.com/rrwick/Rebaler) is another alternative based on a

reference and a long read dataset.

We thank Reviewer #1 for highlighting these methods which have similarity and relevance to our

work. We have updated our background (paragraph 4) to include a description of RACA and

Ragout2 and how they compare and contrast with RaGOO. However, a few major issues

prevented us from directly comparing these methods to RaGOO.

- Firstly, RACA, unlike RaGOO, requires paired-end WGS data in order to perform

scaffolding (https://github.com/ma-compbio/RACA/issues/4). The dependency of pairedend

sequencing for RACA renders it unsuitable for our benchmarking with both real and

simulated data, since these experiments used (non-paired-end read) long read

sequencing data.

- Ragout2 does not require WGS data to scaffold genome assemblies but it depends on

the Cactus multiple sequence aligner for large eukaryotic genomes (> 100 Mpb), which

renders it very inefficient. According to the developers of Cactus, producing alignments

for mammalian sized genomes can take over 100 CPU days

(https://github.com/ComparativeGenomicsToolkit/cactus#systemcluster-requirements).

This is in contrast with RaGOO which, owing to the speed of Minimap2, can scaffold a

human genome in less than 2 core hours (~13 minutes on an 8 core laptop).

Accordingly, we believe that it would be prohibitive to include Ragout2 in our

benchmarking.

- Rebaler is not appropriate for comparison since it is an assembler which works on raw

sequencing reads whereas RaGOO is a scaffolder which works on assembled contigs.

The Rebaler authors also caution that it has only been tested on bacterial genomes, but

has not been evaluated on any eukaryotic genomes (https://github.com/rrwick/Rebaler).

Therefore, we did not include it in our benchmarking or our background discussion.

- ONT data as well as tomato genome assemblies and their annotation should be submitted to

public databases such as NCBI or EBI.

Raw sequencing data for the 3 tomato assemblies described in the manuscript have been

deposited in the NCBI SRA under the accession code PRJNA557253. The assemblies and

associated annotations have been uploaded to the Sol Genomics Network, which serves as the

principal repository for the tomato genome and related species. We are currently working with

SGN administrators to create a public facing URL and expect this will be available within the

next 2 to 3 weeks.

- The algorithm is not sufficiently described in the Methods section, especially in the subsection

related to the chimeric contig correction. It is unclear what are the differences between a real

interchromosomal rearrangement and an interchromosomal chimeric contig, as well as between

a deletion and an intrachromosomal chimeric contig. The authors should add a figure to further

explain the algorithm; Figure 1 is not very useful.

In order to address this, we have added a more detailed description of “chimeric contig

correction” to the methods section (*Description of RaGOO Algorithm and Scoring Metrics*,

paragraph 4) and changed Figure 1 to more clearly illustrate the algorithm. Briefly, chimeric

contig correction, used in the 3 tomato genomes but not the other assemblies in our analysis, is

an optional feature with tunable parameters for expert users, or can otherwise verify that large

structural differences between the assembly and the reference are indeed erroneous.

Potentially intrachromosomal chimeric contigs are broken, by default, if two consecutive

alignments that are greater than 1 Mbp away from each other with respect to the reference. In

theory, this could represent a true 1 Mbp deletion, although in practice such large events are

very rare, especially compared to the rate of mis-assemblies. For example, the 1000 Genomes

project only detected 2 out of 68,818 SVs that were larger than 1 megabase across 2,504

human genomes (Sudmant et al, Nature, 2015, Figure 1b & associated VCF file), and an

analysis of 3 high quality maize genomes (B73, Mo17 and PH207) found that the vast majority

of presence/absence structural variations were less than 5kbp and that only two of the 25,875

variants were over 1Mbp (Sun et al, Nature Genetics, 2018, Supplementary Table 9). Our own

tomato assembly analysis also demonstrates an example where such large-scale structural

differences are unexpected so that chimeric contig correction leads to a more structurally

accurate chromosome-scale assembly (Figure 4).

- Assigning a confident score is an interesting feature. I think the authors should discuss the

scoring method in the main manuscript and show on the genomes of tomato and Arabidopsis

how the score can be useful. "These scores can also be used to assess the efficacy of chimeric

contig correction or to check if an assembly diverges too much from the reference genome for

accurate scaffolding" (P9, L42). I think the authors should show how users can evaluate the

accuracy of the scaffolding using confident scores.

Though the bulk of the description of confidence scores remains in the methods, we expanded

the results section to place more emphasis on this feature in the main text (*Reference-guided*

*Contig Ordering and Orientation with RaGOO*, paragraph 2). We also have added two analysis

components to show-case the practical use of these scores. Firstly, we added supplemental

Figure 2, which shows the confidence score distributions for the M82 genome assembly

scaffolding. This allows readers to compare distributions before and after chimeric contig

correction. It also provides examples of confidence score distributions associated with positive

scaffolding results. Additionally, we demonstrated how confidence scores can be used to

determine if the draft genome assembly is too divergent from the reference genome for optimal

scaffolding results. Our experiment is described in the new section entitled “Scaffolding a

divergent *S. pennellii* genome assembly” (also see below). Supplemental figure 6 shows how

confidence scores will generally decrease when the reference genome is too divergent from the

draft assembly.

- I tested the RaGOO software on several assemblies. On genome assemblies <1G, it runs

pretty fast and the ordering of contigs is reliable. However, I was surprised to find that it does

not produce an AGP file that can be useful for transferring annotations. On a larger genome

(>10 Gb), the alignment step was fast but the software spent 5 days generating

pseudomolecules without providing information, which can be disconcerting for users.

Both of these concerns have already been independently addressed with updates to the

RaGOO software. Now, RaGOO provides a script to produce an AGP file from RaGOO output.

The GitHub commit for this update is here:

https://github.com/malonge/RaGOO/commit/5e505ee80a0263179164bca6c89913400e38801c

The inefficiency described with large genomes was the result of a suboptimal algorithm for

pseudomolecule construction. The optimization addressing this is in the following GitHub

commit.

https://github.com/malonge/RaGOO/commit/1dc1246c6b32bfeb27a8a7e4b2be81800f75f72c

Our new human genome analysis supports our claims that the software is now fast even for

large mammalian genomes.

- I was wondering how RaGOO handles the scaffolding with a more distant reference genome; it

should increase the size of the Chr0 sequence. Another tomato genome has been sequenced

using the nanopore technology, and because it is not the same species (Solanum pennellii,

http://www.plantcell.org/content/29/10/2336) it should be interesting to use it for a test.

We address this in our new section entitled “Scaffolding a divergent *S. pennellii* genome

assembly”. We scaffold the *S. pennellii* contigs from the abovementioned paper with two

different references. One is the same SL3.0 *S. lycopersicum* reference used in our other tomato

analysis and the other is an independent *S. pennellii* reference. If the *S. lycopersicum* reference

is suitable for scaffolding the *S. pennellii* contigs, the two resulting sets of pseudomolecules

should be very similar. However, in supplementary figure 5, we see significant structural

differences between the two sets of pseudomolecules, indicating that the *S. lycopersicum*

reference guided scaffolding masked true chromosomal differences between the two species.

We also observe that a much larger portion (26,868,206 bp vs. 6,230,859 bp) of the *S. pennellii*

contig sequence is unlocalized when using the *S. lycopersicum* reference for scaffolding.

Finally, we show in Supplemental Figure 6 how confidence scores indicated a suboptimal

scaffolding by the *S. lycopersicum* reference.

Note that we remain confident in our *S. pimpinellifolium* scaffolding results since *S.*

*pimpinellifolium* is a closely related progenitor species of *S. lycopersicum*. Indeed, because the

chromosome structure of *S. lycopersicum* and *S. pimpinellifolium* are so similar, it is

straightforward to produce hybrid crosses of the two species, although crosses of *S.*

*lycopersicum* with *S. pennellii* are rarely effective.

- There are big differences in the contiguity of the three tomato genomes, can the authors

explain the reason for these differences. In addition, the assembly of the GBV (sic) genome,

which has the highest contiguity, contains more structural variants than M82 and FLA, do you

think it could be related to the high contiguity of the initial Nanopore assembly?

Assembly contiguity is difficult to predict as it can subtly depend on the exact placement of each

read relative to repeats in the genome. Nevertheless, we attribute the improved contiguity to the

BGV assembly primarily to the fact that it had the longest reads available, with a read N50 of

~27 kbp compared to ~13 kbp and ~24 kbp for M82 and FLA respectively. We do not expect

this to have had a major impact on the SV analysis as the total span of assembled contigs in the

3 assemblies is very similar and the number of SVs found in FLA and BGV are similar. Note that

we expected M82 to have the least number of SVs since it is most similar to the Heinz tomato

reference genome.

- The authors chose to polish the Nanopore consensus after the scaffolding step, but error

correction could improve the minimap2 alignment step. Can the authors comment?

Users have the option of polishing before scaffolding, but we believe this speaks to a utility of

RaGOO since, as you point out, the contigs do not need to be polished to be ordered and

oriented. This is especially convenient if users wish to perform gap-filling after scaffolding so

that polishing only needs to be performed once. Notably, our results show that our tomato

assembly pseudomolecules are accurate and complete, even though contigs were not polished

prior to scaffolding. This is reflected in the relatively small amount (0.5% to 1%) of sequence

that was unlocalized and placed in the chromosome 0, which we attribute to the incomplete

nature of the tomato reference genome. RaGOO also localizes ~99.9998% of the “hard”

scaffolds in our simulated experiments, demonstrating that minimap2 alignments are sensitive

enough to localize gapped sequence that is variable with respect to the reference genome (1%

SNP rate + SVs).

- A gap-filling step was performed using PBJelly but a previous study found that PBJelly

generates artefactual duplications (https://www.nature.com/articles/sdata2018235). It would be

good to check these artifacts as they can lead to the prediction of false structural variants.

We include a new analysis in our methods section to assess this (*Tomato Genome Correction*

*and Polishing*, paragraph 4). First, we called structural variants in our gap-filled assemblies

(before polishing) with respect to the Heinz SL3.0 reference genome using Assemblytics. We

then found all “Tandem_expansions” (duplications) that occur in gaps filled by PBJelly. All but

one variant in M82 were supported by read coverage when aligning the raw ONT data to the

gap filled assemblies. For t he one potentially spurious duplication, we found its exact match

in the final polished M82 assembly and indicate the coordinates. We do not believe that it is

warranted to modify the final assemblies on account of this analysis, but we provide the

potentially spurious coordinates for reference.

Minor points:

- Was the chimeric contig correction turned on for the scaffolding of the Arabidopsis

assemblies? I cannot find the information in the manuscript.

Chimeric contig correction was not used. This has been updated in the methods (*Arabidopsis*

*Structural Variant Analysis*, paragraph 1).

- The Supplementary Tables are either missing or poorly formatted in the pdf file I received.

We believe we have fixed this issue with the current submission.

- P2: "Acquiring a genomic map could be expensive". Two recent studies have reported

chromosome-scale assemblies based on long reads and optical maps with reasonable costs

(10.1038/s41477-018-0289-4 and 10.1038/s41467-018-07271-1).

We agree that Bionano maps have become less expensive for scaffolding draft assemblies.

Based on recent quotes we have for scaffolding novel genomes of similar size optical mapping

costs several thousand dollars per genome when including labor, sample preparation, and

imaging. This may be considered relatively cheap or expensive depending on the project.

However, we believe that our statement is ultimately fair, especially when generally referring to

“genomic maps”, including genetic maps, and especially when comparing to reference-guided

scaffolding, which is essentially free.

- P3: "can optionally avoid breaking chimeric intervals at loci within…". I think this is an

interesting feature, because it will allow the scaffolding of already annotated assemblies.

Yes, that was our goal. Thank you for your feedback.

- P5: Please provide the standard metrics of the SALSA2 assembly.

These metrics have been added to the methods section (*Reference-Guided and Reference-*

*Free Scaffolding of Tomato Genomes*, paragraph 2).

- P8: "… to uncover genomic variation not discovered through traditional short-read mapping".

The authors must prove that these genomic variations could not be discovered using short

reads or delete this sentence.

While this result has been seen in several other projects, we have changed the language in this

section and no longer make this claim.

- P13: "BGV underwent two rounds of chimeric contig correction.". Why is the process different

for the assembly of the BGV genome?

This was a decision based on an inspection of the corrected pseudomolecules. Sometimes

multiple rounds are necessary because contigs can only be broken once per round. Though

scaffolding was sufficient for FLA and M82 after one round, two rounds were needed for BGV to

correct all of the apparent misassemblies. This is now reflected in the methods section

(*Description of RaGOO Algorithm and Scoring Metrics*, paragraph 5).

========================================================================

Reviewer #2: The paper is devoted to a newly developed open-source tool RaGOO which is

intended to accurately reconstruct

contig ordering and orientation guided by a reference genome. RaGOO (based on Minimap2

aligner) is very fast in comparison with available tools

(~1B bp plant genome is processed in 7 min).

There two types of results - validation of the tool and report on newly

identified structural variations in plant genomes.

In general, I believe that application to plant genomes may be more stressed in conclusions -

it is not clear how well it will performed on animal genomes. Specifically, the main competitor is

Chromosomer which was originally applied to insects.

RaGOO has been successfully applied to tomato genome (M82 reference cultivar).

Validation of scaffolding results using de novo assemblies of

Oxford Nanopore long-read sequencing is a significant advantage of the study.

It allowed show that the tool is generally very accurate.

Numerous structure variations

which were not recorded before have been identified for M82.

A pan-genome analysis of 103 Arabidopsis

thaliana accessions also identified newly detected structural variations.

- The methods are appropriate and well described.

- Conclusions are adequately supported by results and shown data.

- Sufficient details are provided to allow replication and comparison with related analyses.

- The software (available at github) has been compared with existing tools (Chromosomer and

show-tiling) and show advantages both in quality and efficiency.

It would be advantageous to apply to non-plant genomes - although i would not require that for

the publication.

Thank you for your suggestion and your positive comments. We have added an analysis

describing the scaffolding of human genome in the results (*Reference-guided Contig Ordering*

*and Orientation with RaGOO*, paragraph 3) and methods (*Scaffolding of an Arabidopsis thaliana*

*and human genome*). This shows how we are able to scaffold a human genome in ~13 minutes,

and report on the alignment of the resulting high quality pseudomolecules to the human

reference genome (Supplementary Figure 1).

The paper is of broad interest to bioinformaticians specialized in sequence assembly as well as

to biologists who assemble large genomes especially plant genomes.

The paper and the results including a new annotated release of M82 tomato genome will be of

particular interest to plant biologists. The tool can be used by a very

broad audience for assembly of large genomes, finding large structural variations, and

annotating pan-genomes.

Minor comments:

- mention that validation are on plant genomes.

Especially now that we have added our human genome analysis, we believe that RaGOO can

justifiably be described as a tool for eukaryotic genomes in general. Our general focus on plants

is a result of data availability and research interests. However, there is no plant-specific design

component of RaGOO. For example, the default Minimap2 alignment parameters are generic,

and can work for a wide variety of eukaryotic genomes.

- Supplementary Table 1 is missing?

We hope to have addressed this with the current submission.

- Supplementary Table 3 is impossible to read since it is partitioned between pages and there is

no pointer to excel

We hope to have addressed this with the current submission.

**Second round**

**Reviewer 1**

The authors present an improved manuscript “Fast and accurate reference-guided scaffolding of draft genomes” and my major compulsory revisions were taken into account.
